# Development of Supramolecular Calix[4]arene-MCM-Based Nanomaterials Doped with CuNPs: An Inorganic–Organic Hybrid Recyclable Nanoenzyme as a Potent Peroxidase and Oxidase Mimic

**DOI:** 10.1021/acsomega.6c07489

**Published:** 2026-10-16

**Authors:** Meenakshi Goyal, Anuj Kumar, Pardeep Kumar, Bhawna Uttam

**Affiliations:** † Department of Chemistry, J.C. Bose University of Science and Technology, YMCA, Faridabad, Haryana 121006, India; ‡ Department of Chemistry, Indian Institute of Technology Bombay, Powai, Mumbai 400076, India; § Department of Chemical Engineering, 2358 University of Virginia, Charlottesville, Virginia 22904, United States

## Abstract

An inorganic–organic hybrid nanomaterial, Cu@MCMCal, was synthesized by immobilizing *p*-*tert*-butyl calix[4]­arene on to mesoporous silica followed by incorporation of CuNPs. The synthesized nanomaterial was characterized extensively using various analytical techniques such as FT-IR, UV–Vis spectroscopy, TEM, PXRD, XPS, Cu LMM Auger, BET, and TGA analyses. The hybrid nanomaterial was evaluated for its catalytic performance toward peroxidase-like activity using 3,3′,5,5′-tetramethylbenzidine (TMB) and *o*-phenylenediamine (OPD) as a substrate at room temperature at pH 4.5 in acetate buffer and pH 7.4 in PBS buffer, respectively. Enzyme kinetics was performed for both the substrates using the Michaelis–Menten steady-state kinetics and the Lineweaver–Burk plot to determine the catalytic affinity. Based on the kinetic measurements, the values of the *K*_m_ for TMB and OPD were found to be 0.661 ± 0.143 and 0.606 ± 0.061 mM, respectively. The hybrid nanoenzyme also exhibits the oxidase-like activity using dopamine as a substrate at pH 7.4 in PBS buffer. The Cu@MCMCal nanomaterial acted as a nanoenzyme, efficient for catalysis toward oxidation reactions with better cost effectiveness and improved stability and recyclability as compared to biological enzymes. Therefore, a promising, stable, efficient, and robust nanoenzyme has been development for oxidation reactions.

## Graphical abstract

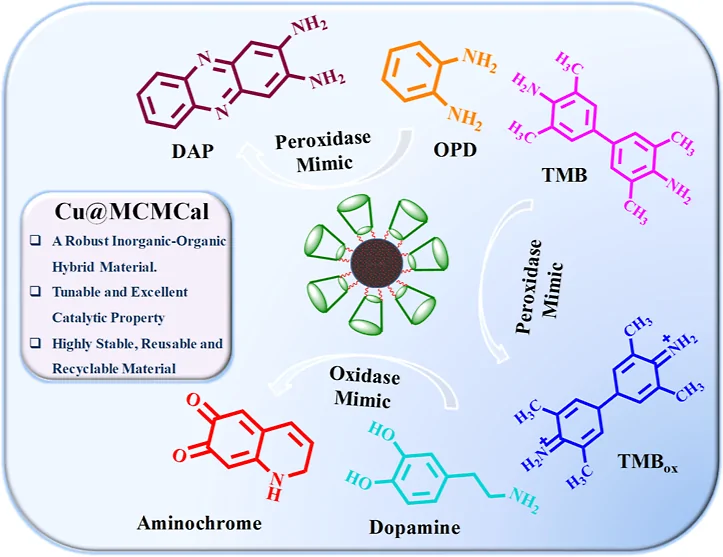

## Introduction

Supramolecular hosts have been extensively used as an efficient candidate for multidisciplinary research. The various types of supra-molecular hosts, such as calix­[*n*]­arene, pillar­[*n*]­arene, cucurbit­[*n*]­uril, cyclodextrin, crown-ethers, and many others, have been explored in the area of ion-sensing, catalysis, molecular recognition, drug delivery carriers, and gene and photodynamic therapies.^1–4^ The supramolecular systems have predominant noncovalent interactions, such as electrostatic, hydrogen bonding, π–π stacking, van der Waals, and hydrophilic/hydrophobic interactions.^5–7^ Among these different supramolecules, “calix­[*n*]­arene” has been found to be a potent candidate for a wide variety of applications. Calix­[*n*]­arenes are the cyclic oligomers composed of phenolic units linked by methylene (−CH_2_−) bridges, forming a flower vase-like structure with an inner hydrophobic core and modifiable upper and lower rims. The hydrophobic core of the calix­[*n*]­arene facilitates in guests binding through the noncovalent interactions. The modifiable lower and upper rims resulted in a wide variety of amphiphilic supramolecules and thereby enhancing their versatility toward different applications.^8,9^

The calix[4]­arene macrocyclic hosts are found to be selective and efficient sensors for a variety of metal ions, anions, amino acids, pollutants, etc. and have also been used as a potent candidate as drug delivery vehicles, for cell transfection, and in the area of catalysis.^1,10,11^ Calixarene derivatives have also been explored as a heterogeneous catalyst by anchoring on to the various inorganic materials and solid supports, such as activated carbon, MOFs, alumina, silica, etc., resulting in the formation of inorganic–organic hybrid materials.^12,13^ Among the variety of inorganic supports, mesoporous silica (MCM-41) features a well-ordered hexagonal array of cylindrical mesopores, with pore sizes ranging from 2 to 10 nm with typical pore sizes in the range of 2–4 nm and an exceptionally high surface area exceeding 1000 m^2^/g. Due to remarkable properties, such as tunable pore size, large pore volume, excellent thermal and chemical stability, and ease of functionalization with various chemical groups, mesoporous silica has been found to be a potent inorganic support. The covalent functionalization of MCM-41 with organic host molecules, such as calix[4]­arenes resulted in an inorganic–organic hybrid that has been extensively studied for various applications, including heavy metal adsorption, separation processes, catalysis, and drug delivery.^14–17^ The incorporation of metal nanoparticles (NPs) in such an inorganic–organic hybrid resulted in the formation of nanomaterials suitable for catalytic applications. Among different metal NPs, copper nanoparticles (CuNPs) have several advantages over Au, Ag, Pt, and Pd NPs due to their high natural abundance, cost-effectiveness, excellent catalytic activity, high stability, and redox properties. CuNPs have been encapsulated in various frameworks like MOFs, nanoflowers, silica, zeolites, mesoporous carbon nitride, graphene oxide, and carbon composites and explored for various types of catalysis such as oxidation, reduction, C–C coupling, enzyme mimic, detection and sensing applications, electrocatalysis, and water remediation.^18–30^ On the other hand calix­[*n*]­arene derivatives doped with metal NPs have also been explored for a variety of applications. Calix[4]­arene phosphine ligands have been explored as a macrocyclic mimic of protein backbone for stabilizing the gold nanocluster.^31^ The *p*-sulfonato calix[4]­arene-based nanoenzyme has been developed and explored for the detection of H_2_O_2_ in the living cell.^32^ However, the literature reports on the calix[4]­arene-based inorganic–organic hybrid nanomaterials as a nanoenzyme for enzyme mimic activity are scarce. In spite of having high substrate specificity and high catalytic efficiency, the natural enzyme has several limitations such as high cost, purification challenges, heterogeneity, and recyclability. Therefore, there is a dire need to develop a cost efficient, highly stable, and heterogeneous hybrid material that can potentially work as nanoenzymes.

The CuNPs undergo nanoparticle agglomeration, surface oxidation, and loss of active sites due to repeated catalytic cycles. However, the dispersion of these nanoparticles into MCM-41 resulted in suppression of nanoparticle agglomeration and gave structural stability but lower substrate affinity. To address this, in the current study, an inorganic–organic nanohybrid material doped with CuNPs has been designed and developed. The design strategy is such that (i) CuNPs act as both catalytic and redox-active centres due to their ability to undergo reversible changes in the oxidation state, (ii) MCM-41 provides the inorganic support and makes the nanomaterial heterogeneous along with the surface area and center for the covalent functional itself, and (iii) the incorporation of *p*-*tert*-butylcalix­[4]­arene onto the mesoporous silica framework enhances the stability and catalytic efficiency of CuNPs by preventing aggregation and leaching. The hydrophobic cavity of calix[4]­arene provides a favorable environment for substrate binding by enhancing their accessibility to the reaction center through the noncovalent interactions. This synthesized nanohybrid material, i.e., Cu@MCMCal, was explored as a nanoenzyme toward peroxidase- and oxidase-like activities using 3,3′,5,5′-tetramethylbenzidine (TMB), *o*-phenylenediamine (OPD), and dopamine as substrates.

## Methods and Materials

The chemicals 3-glycidyloxypropyl-trimethoxysilane (GPTMS), tetraethyl orthosilicate (TEOS), cetyltrimethylammonium bromide (CTAB), copper sulfate pentahydrate (CuSO_4_·5H_2_O), poly­(vinyl alcohol) (PVA), ascorbic acid, 3,3′,5,5′-tetramethylbenzidine (TMB), o-phenylenediamine (OPD), dopamine hydrochloride, hydrogen peroxide (H_2_O_2_), isopropyl alcohol (IPA), disodium ethylenediaminetetraacetic acid (Na_2_EDTA), and *p*-benzoquinone (PBQ). All these chemicals were procured from Sigma-Aldrich and CDH.

FTIR analysis was conducted on a PerkinElmer Spectrum TWO L1600300 LiTa instrument at a resolution of 0.5 cm^–1^. UV–Vis absorption measurements were carried out using a Shimadzu UV-3600i PLUS spectrophotometer. X-ray diffraction (XRD) studies were performed using a PANalytical Empyrean X-ray diffractometer equipped with a Cu target X-ray tube operating at a wavelength of 1.54184 Å. Thermogravimetric analysis (TGA) was conducted on a PerkinElmer Diamond TG/DTA Instruments with a heating rate range of 0.01–100 °C. Transmission electron microscopy (TEM) was performed using a TecnaiG20 high-resolution TEM (200 kV) at SAIF, AIIMS, and X-ray photoelectron spectroscopy (XPS on a Kratos Analytical AXIS Supra instrument. The BET analysis measurements were done on BELSORP-maxII (BELSORP series) by Microtrac BEL Corp. The SAXS measurements were recorded on a Xenocs Xeuss 2.0 instrument using a GeniX3D Cu microfocus X-ray source (λ =1.54 Å) operating at 50 kV/0.6 mA).

### Synthesis of Mesoporous Silica

250 mg of CTAB was added to 120 mL of water followed by 0.875 mL of NaOH and the resulting solution was stirred at 60 °C for 13 min to obtained a homogeneous solution. To this, 5 mL of TEOS was added dropwise with continuous stirring followed by aging for 2 h. The resulting solution was centrifuged at 8000 rpm for 15 min followed by extensive washing with distilled water to remove excess of CTAB. The crude solid was dried at room temperature followed by calcination at 555 °C for 5 h to obtain MCM-41.^33^

### Synthesis of calix[4]­arene

The *p*-*tert*-butyl calix[4]­arene has been synthesized using a literature-reported method.^17^

### Synthesis of MCMCal Hybrid

*p*-*tert*-Butylcalix­[4]­arene (100 mg, 0.15 mmol) and GPTMS (74 μL, 0.33 mmol) were added to 15 mL of dry toluene followed by the addition of three drops of perchloric acid. The resulting reaction mixture was then heated at 80 °C for 15 h under nitrogen atmosphere to derivatize alternate the OH at the lower rim. To this 500 mg of MCM-41 was introduced into the reaction mixture followed by refluxing at 110 °C for 48 h. The resulting crude product (MCMCal) was collected by centrifugation at 8000 rpm followed by washing with toluene, dichloromethane, water, methanol, and acetone to remove the excess of reactant impurities. Finally, inorganic organic hybrid was dried under vacuum for 2 h, yielding 394 mg (58%) of MCMCal.^17^

### Synthesis of CuNPs

Copper nanoparticles (CuNPs) were synthesized using a reduction method. To one gram (4 mmol) of CuSO_4_·5H_2_O (dissolved in 50 mL of deionized water) was added 0.1 g of poly­(vinyl alcohol) (PVA) (in 20 mL of distilled water), and the mixture was stirred continuously for 30 min. To the resulting solution was added 0.5 g (2.8 mmol) of ascorbic acid in 50 mL of distilled water dropwise with continuous stirring at room temperature. Initially, the solution color changed from blue to pale green and finally to red, confirming the successful formation of CuNPs. Then, the resulting solution was centrifugated at 8000 rpm for 15 min and washed multiple times with deionized water followed by ethanol to remove unreacted starting materials. The resulting CuNPs were dried under vacuum for 12 h and further characterized by TEM, PXRD, and XPS.^34,35^

### Synthesis of Cu@MCMCal

Cu@MCMCal was synthesized by incorporating Cu nanoparticles (CuNPs) into MCMCal. To 100 mg of MCMCal was added 20 mg of CuNPs, which were continuously stirred for 24 h in ethanol. The resulting nanomaterial, Cu@MCMCal, was isolated by centrifugation at 8000 rpm followed by multiple washings and drying for 5 h.

### Peroxidase Mimic Activity of Cu@MCMCal

The peroxidase-mimic activity of Cu@MCMCal was investigated using the substrates, TMB and OPD. The stock solutions of 6 × 10^–3^ M concentration of TMB and OPD were prepared in ethanol and deionized water, respectively. The oxidation of TMB by the Cu@MCMCal was studied by using H_2_O_2_ at pH 4.5 in acetate buffer. Kinetic measurements were performed using TMB concentration from 0.1 to 2.0 mM, with a fixed H_2_O_2_ concentration of 80.6 mM (25 μL of 30% w/w of H_2_O_2_) and Cu@MCMCal as 50 μg/mL. In another experimental setup, the H_2_O_2_ concentration was varied from 10 to 326.6 mM and keeping the TMB and Cu@MCMCal catalyst concentration constant as 0.5 mM and 50 μg/mL, respectively. The OPD concentration was varied from 0.1 to 4 mM keeping the H_2_O_2_ concentration constant as 50 mM (15 μL of 30% w/w of H_2_O_2_) in the presence of 50 μg/mL of Cu@MCMCal in PBS buffer (10 mM, pH 7.4) at room temperature. In another set of experiments, H_2_O_2_ concentration was varied from 0.33 to 10 mM keeping the OPD and catalyst concentration constant as 0.5 mM and 50 μg/mL, respectively, in 10 mM of PBS buffer (pH 7.4) at room temperature. The absorption spectra were recorded up to 12 min at different time intervals. Kinetic parameters of Cu@MCMCal were evaluated by using Michaelis–Menten equation (eq 1) and further extended to the Lineweaver–Burk equation (eq 2), where V is the initial reaction velocity, *V*_max_ is the maximum reaction velocity, [*S*] is the substrate concentration, and *K*_m_ is the Michaelis–Menten constant. The value of *K*_m_ represents the substrate concentration at which the rate of enzyme catalysis reaction becomes half of the maximum velocity. The values of *K*_m_ and *V*_max_ were determined from the Michaelis–Menten equation.

1 $$V=\frac{\left(V_{\max}\left[S\right]\right)}{\left(K_{m}+\left[S\right]\right)}$$

2 $$\frac{1}{V}=\left(\frac{K_{m}}{V_{\max}\left[S\right]}\right)+\frac{1}{V_{\max}}$$

### Oxidase-Like Activity

The oxidase-like activity of Cu@MCMCal nanoenzyme was studied using dopamine hydrochloride as a substrate. The stock solution of 1 mM of dopamine hydrochloride was prepared by dissolving 1.9 mg of dopamine hydrochloride in 10 mL of solvent. The oxidation of dopamine (80 μL conc.) was studied by UV-absorption spectroscopy for 50 min in 10 mM of PBS buffer (pH 7.4) in the presence of 50 μg/mL of Cu@MCMCal catalyst.

### Recyclability Studies

The recyclability of Cu@MCMCal nanoenzyme was carried out for few consecutive catalytic cycles. One milligram of Cu@MCMCal was dispersed in a 3 mL quartz cuvette containing 300 μL of TMB stock solution (0.6 mM) followed by addition of 25 μL of H_2_O_2_ (30% w/w). Upon completion of each run, the nanoenzyme was separated by centrifugation at 8000 rpm for 10 min followed by thorough washing with deionized water and ethanol. The precipitate of the nanoenzyme was suspended again in water and reused for next consecutive cycles. After each run, the nanoenzyme was recovered by following the same procedure.

### Scavenging Experiment

To identify the reactive oxygen species (ROS) generated during the peroxidase-like activity of Cu@MCMCal nanoenzyme during the oxidation of TMB in the presence of H_2_O_2_ was investigated by performing the scavenging experiments. IPA, PBQ, and ethylene diamine tetracetic acid (EDTA) were used as a scavengers for the quenching of ROS as a hydroxyl radical (^•^OH), superoxide radical (O_2_^•–^), and holes (h^+^), respectively. The absorption studies were carried out using 0.2 mM TMB with 25 μL of 30% (w/w) H_2_O_2_ in the presence of 50 μg/mL of Cu@MCMCal nanoenzyme. The concentrations of the scavengers used were 10 μL of 99.5% IPA and 10 mM for both PBQ and EDTA.

## Results and Discussion

The inorganic–organic hybrid nanomaterial, i.e., MCMCal, was synthesized by reacting *p*-*tert*-butyl calix[4]­arene and 3-glycidyloxypropyltrimethoxysilane in the presence of dry toluene at 80 °C. The resulting hybrid nanomaterial was doped with CuNPs to form a robust nanoenzyme, Cu@MCMCal, as can be observed from Scheme 1.

**Scheme 1:**
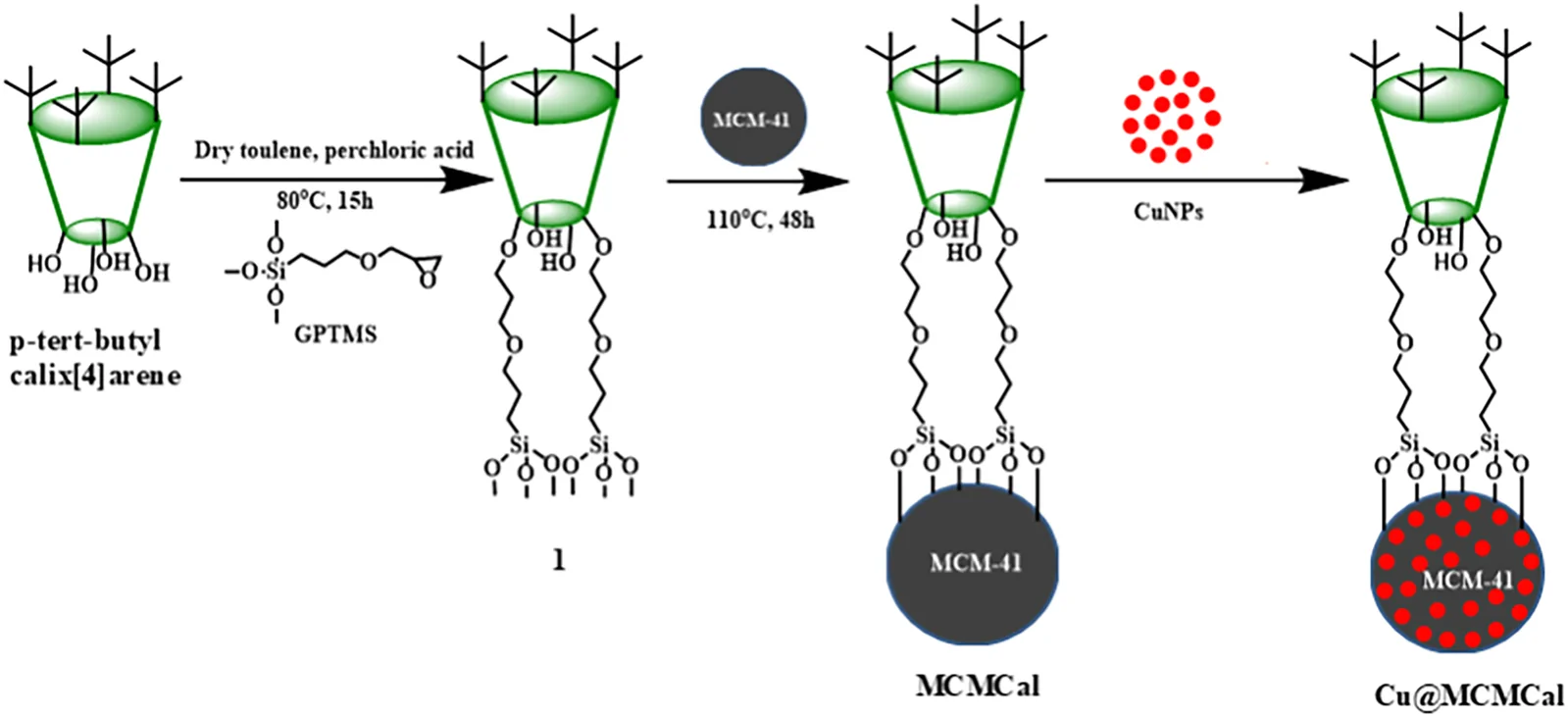
Synthetic Route for the Synthesis of Inorganic–Organic Hybrid Material, Cu@MCMCal

The Cu@MCMCal nanomaterial was characterized by various spectroscopic and microscopic techniques such as UV–Vis, FTIR, TEM, PXRD, TGA, XPS, and BET. The FT-IR spectrum of MCM-41 exhibits distinct vibrational peaks. The strong stretching peaks appeared at 1066 and 805 cm^–1^ corresponding to the asymmetric and symmetric stretching vibration of the Si–O–Si bonds, respectively. Additionally, a peak at 456 cm^–1^ is associated with the bending vibration of the Si–O–Si bonds. A broad peak was also observed at 3410 cm^–1^, which corresponds to the stretching vibration of the O–H bond in the silanol group (Si–OH). Furthermore, the peaks at 959 and 1628 cm^–1^ are due to the bending vibration of the Si–OH bond and vibration of water molecules, respectively. The FT-IR spectrum of *p*-*tert*-butylcalix­[4]­arene shows characteristic peaks at 3144 cm^–1^ attributed to the stretching vibration of the phenolic −OH and at 2955, 2886, and 1700 cm^–1^ are associated with aromatic and alkyl −CH stretching and aromatic C=C stretching vibrations, respectively. MCMCal exhibits all the characteristic peaks of MCM-41, along with the additional peaks corresponding to asymmetric and symmetric vibrations of −CH_2_ groups supporting the successful immobilization of *p*-*tert*-butylcalix­[4]­arene onto MCM-41­(Figure 1a). The FTIR spectrum of CuNPs can be shown from Figure S01b.

**Figure 1:**
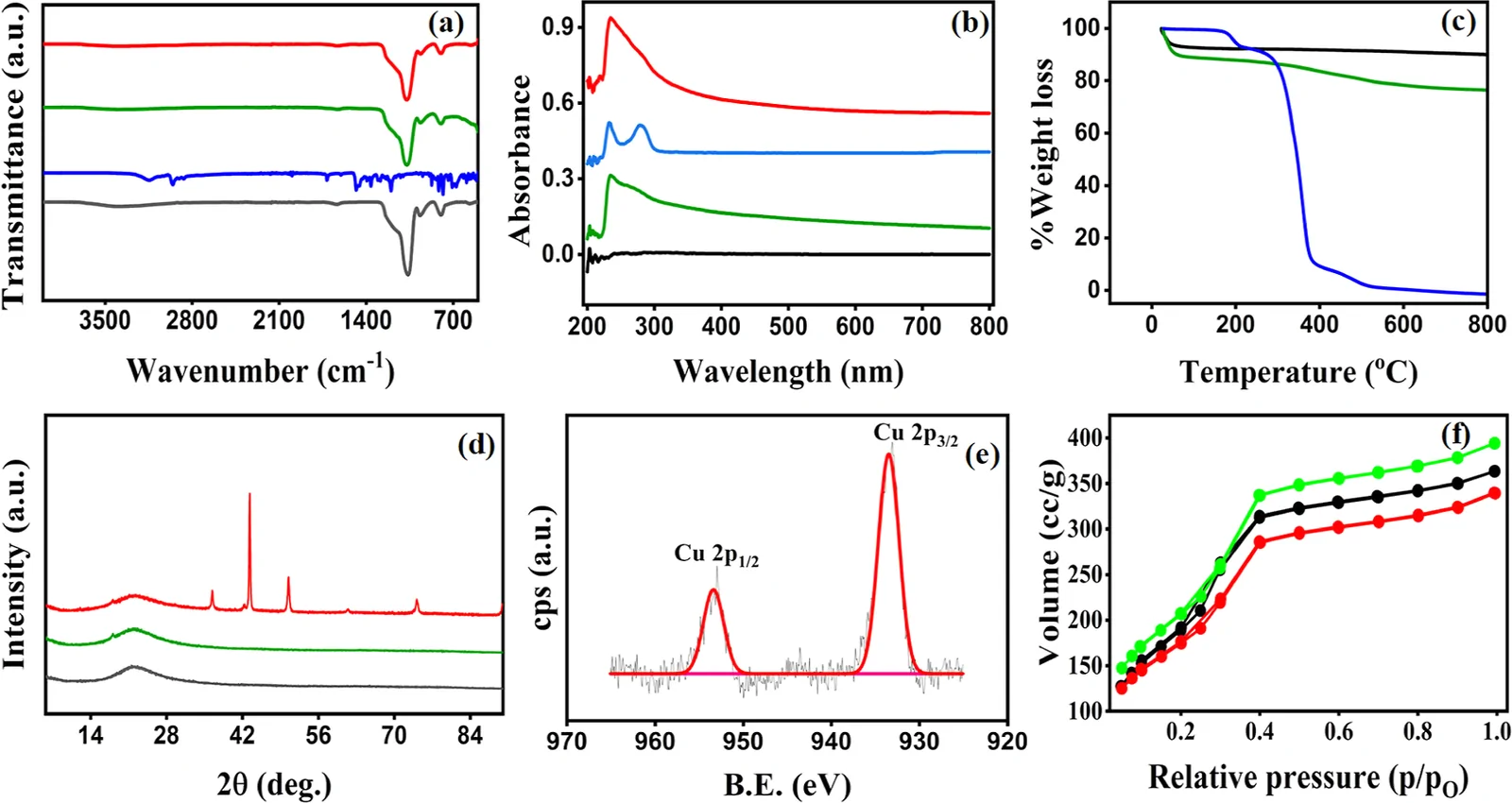
Characterization data of Cu@MCMCal hybrid nanoenzyme. (a) FTIR; (b) UV–Vis; (c) TGA; (d) PXRD; (e) XPS; and (f) BET; color code MCM-41 (black); MCMCal (green); Cu@MCMCal (red); and calix[4]­arene (blue).

The electronic spectrum was recorded for calix[4]­arene and calix[4]­arene on MCM-41 to elucidate the structural information. The UV absorption spectrum of calix[4]­arene exhibits two distinct absorption peaks at λ = 232 and 279 nm corresponds to π → π* transition associated with benzene rings and *n* → π* transition due to the presence of phenolic groups in the calix[4]­arene, respectively. However, the MCM-41 does not show any significant absorption peaks in the UV–Vis region due to the absence of electronic transitions within the Si–O–Si network. However, the MCMCal hybrid nanomaterial spectrum retains both the absorption band of calix[4]­arene, suggesting the immobilization of calix[4]­arene on MCM-41 (Figure 1b).

MCM-41 exhibited ∼6.39% weight loss at 100 °C due to removal of adsorbed water on the surface. The subsequent gradual weight loss up to 290 °C in MCM-41 is due to the condensation of silanol groups forming siloxane bonds. The calix[4]­arene showed a significant decomposition of 90.67% up to the temperature 400 °C. However, MCMCal was found to be very robust and stable as compared to calix[4]­arene and exhibits 23% of weight loss when heated up to 700 °C (Figure 1c), corresponding to the decomposition of organic moiety, i.e, calix[4]­arene. This data suggests the ∼13% loading of the calix[4]­arene on to the MCM resulting in the inorganic–organic hybrid material. The CuNP loading in Cu@MCMCal was determined by ICP–MS. The analysis showed that 7.43 wt % of Cu NPs was successfully loaded in the MCMCal framework.

Structure morphology and crystallinity of the hybrid material were further investigated by X-ray crystallography. The PXRD peaks for CuNPs observed at 2θ = 43.28°, 50.33°, and 74.5° correspond to the (111), (200), and (220) planes of face-centered cubic, confirming that copper exists predominantly in the Cu^0^ state in CuNPs (Figure S01a).^36^ The low-angle PXRD of MCM-41 contains a sharp peak at 2θ = 2.12° at (100) reflection and Braggs peaks at (110), (200), and (210) at 2θ = 4.15°, 4.80°, and 6.35° correspond to the hexagonal array of MCM-41. By using the Bragg’s law (nλ = 2dsinθ), the calculated value of *d*_100_ = 4.164 nm and corresponding hexagonal unit cell parameter was *a*_o_ = 4.808 nm. These are the characteristic parameters for the hexagonal array of MCM-41. The low-angle PXRD of MCMCal and Cu@MCMCal contained all the characteristics low-angle reflection associated with MCM-41. The retention of these reflections even after immobilization of calixarene and incorporation of CuNPS indicates that the hexagonal mesoporous arrangement of MCM-41 did not undergo any significant change. However, a decrease in the intensity and resolution of the characteristic Bragg reflections was observed after immobilization of calixarene. This decrease in intensity is generally observed upon organic functionalization of MCM-41 and can be attributed to the incorporation of organic calixarene moieties within the mesopore channels (Supporting Information, Figure S10 and SI10). The broad peak at 22.05° in MCM-41 is attributed to the amorphous silica framework. In the case of MCMCal and Cu@MCMCal, a similar peak (22.54°) with a slight shift was observed, which indicates the retention of the amorphous silica framework in MCMCal. The presence of peaks at 2θ = 43.22°, 50.40°, and 74.5° in Cu@MCMCal supports our claim of effective and successful incorporation of CuNPs in the inorganic–organic hybrid nanomaterial, Cu@MCMCal. Two additional peaks were also observed at 2θ = 36.49° and 61.43° correspond to (111) and (220) planes of Cu_2_O suggesting the CuNPs are in a mixed oxidation state (Cu^0^/Cu^1+^) in Cu@MCMCal, which was further supported by X-ray photoelectron spectroscopic measurements (XPS) (Figure 1d).^37^ The XPS data of the CuNPs alone exhibited peaks at 934.03 and 953.99 eV corresponding to Cu 2p_3_/_2_ and Cu 2p_1_/_2_, respectively, accompanied by their satellite peaks at 942.06 and 962.42 eV (Supporting Information, Figure S01e and SI01). These characteristic features are typical for Cu^2+^ species in copper oxide phases.^36^ However, the XPS peaks of Cu 2p_3_/_2_ and Cu 2p_1_/_2_ in the inorganic–organic hybrid nanomaterial, i.e., Cu@MCMCal, were shifted at 933.48 and 953.37 eV, respectively, with negligible satellite peaks (Figure 1e). This suggests the partial reduction of Cu^2+^ to Cu^+^/Cu^0^, facilitated by the electron-donating phenolic and aromatic groups in calix[4]­arene. The corresponding XPS data for various elements present in Cu@MCMCal can be noticed from Supporting Information Figure S02 and SI02. The Cu LMM Auger spectrum of Cu@MCMCal further supported the coexistence of different oxidation states of copper (Supporting Information Figure S03 and SI03). The Cu LMM Auger spectra contained peaks at 916.1 and 918.7 eV, which correspond to Cu^+^ and Cu^0^ species, respectively. The broad peak at 917.4 eV is associated with the overlapping of Cu LMM contributions of the oxidized Cu species. The analysis of Cu LMM Auger and Cu 2p XPS collectively validates the presence of Cu^0^ and Cu^+^ in Cu@MCMCal, while the presence of Cu^2+^ can be due to the surface oxidation of CuNPs. Based on the experimental results of PXRD, XPS, and Cu LMM Auger, in the Cu@MCMCal nanomaterial, Cu coexists in different oxidation states, Cu(0)/Cu­(I)/Cu (II), and will undergo redox reaction during the catalysis. As PXRD is a bulk-sensitive technique with a penetration depth in the micrometer range and therefore cannot detect minor surface oxidation, whereas XPS probes only the top 5–10 nm of the surface and is highly sensitive to surface chemical states. Therefore, the Cu oxidation state in CuNPs is Cu^0^ as per PXRD (in bulk) and Cu^2+^ (on the surface) as revealed by XPS. Overall, in the hybrid nanomaterial, Cu@MCMCal, the oxidation state of Cu is Cu^+^/ Cu^0^.

The BET data revealed that all the nitrogen adsorption–desorption corresponds to the MCM-41, MCMCal, and Cu@MCMCal follows the type-IV isotherm with a hysteresis loop corresponding to the mesoporous nature of the materials. The absorption isotherm shows a sharp inflection at moderate relative pressure 0.2–0.4 (p/p_o_) due to capillary condensation within the mesopores of materials. The surface area of MCM-41 was found to be ∼793 m^2^/g, which was increased up to ∼800 m^2^/g upon immobilization of calix[4]­arene on MCM-41. The doping of CuNPs in the MCMCal led to the surface area was decreasing up to ∼680 m^2^/g. The average pore size of MCM-41 was observed to be ∼2.8 nm, which increased to ∼3.1 nm in the case of MCMCal 41 further decreased to ∼3.0 nm after Cu incorporation. The change in the surface area and pore diameter on incorporation of CuNPs in MCMCal due to the partial incorporation of CuNPs in the mesopores of MCMCal (Figure 1f). The TEM analysis showed that the average particle size of CuNPs was ∼2–4 nm, comparable to the pore diameter of MCMCal (∼3.1 nm). The porous nature of Cu@MCMCal nanomaterial is beneficial for the catalytic activity and will be helpful in rapid substrate diffusion through the porous framework.

The structure morphology and the size of Cu@MCMCal nanomaterial was analyzed by TEM microscopy. TEM of MCM-41 exhibits well-dispersed NPs with an average size of 200–250 nm as can be noticed from Figure 2a,b,e, clearly showing the porous nature of MCM-41. Upon immobilization of calix[4]­rene on MCM-41, no significant changes in morphology were observed confirming the intact nature of the mesoporous structure (Figure 2c,d,o). The TEM images and particle size distribution of CuNPs (Figure 2f,j) reveal well-dispersed NPs with an average size between 2–4 nm. Furthermore, the TEM micrograph of Cu@MCMCal confirms the incorporation of CuNPs within the MCMCal framework as validated by HAADF-STEM micrographs showing the presence of glowing metal in inorganic–organic hybrid nanomaterials (Figure 2i–k). The EDX mapping of Cu@MCMCal further confirmed the presence of all the elements in the hybrid material (Figure 2l–n).

**Figure 2:**
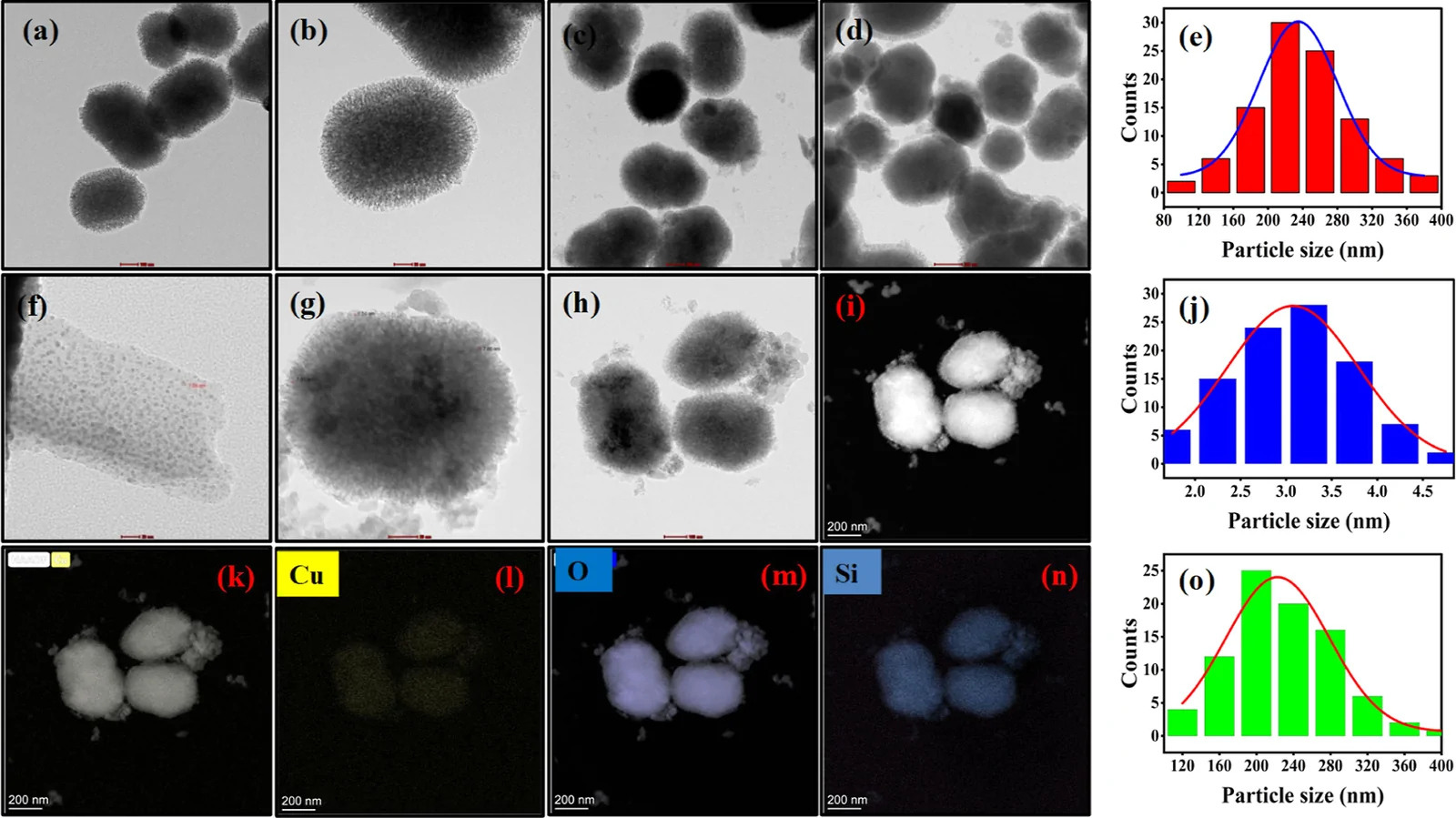
TEM micrographs: (a,b) MCM-41; (c,d) MCMCal; (e) particle size distribution of MCM-41; (f) CuNPs; (g,h) Cu@MCMCal; (i) HAADF–STEM of Cu@MCMCal; (j) particle size distribution of CuNPs; (k–n) shows the EDX mapping TEM micrograph of Cu@MCMCal as per the labels; and (o) particle size distribution of MCMCal.

The inorganic–organic hybrid nanomaterial, Cu@MCMCal, has been successfully characterized by various analytical, spectroscopic, and microscopic methods. The obtained hybrid material was further explored toward various oxidation reactions.

### Peroxidase-Like Activity

The peroxidase-mimicking activity of Cu@MCMCal nanoenzyme was studied by using the substrate TMB and OPD in the presence of H_2_O_2_ (Figure 3a). The oxidation of TMB was carried out in acetate buffer (pH 4.5) at room temperature. The Cu@MCMCal nanomaterial showed peroxidase-like behavior by oxidizing TMB to TMB_ox_ (an oxidized form). The oxidized product (TMB_ox_) was monitored by UV–Vis spectroscopy by measuring the absorbance at λ_max_ = 652 nm and its formation was indicated by a gradual increase in the intensity of blue color in the reaction mixture. The oxidation of TMB in the presence of nanoenzyme, Cu@MCMCal, and H_2_O_2_ was monitored for 12 min with a time interval of 2 min as depicted in absorption spectra, Figure 4b. The gradual increase in the intensity of the blue color (TMB_ox_) with time can be depicted from Figure 3d. To investigate the role of the catalyst and H_2_O_2_, the control experiment was carried out using a TMB substrate. Only the catalyst (nanoenzyme) does not show any peroxidase-like behavior without the presence of H_2_O_2._ The oxidant alone (H_2_O_2_) showed ∼6% of the peroxidase activity as compared to the nanoenzyme, i.e., Cu@MCMCal, in the presence of H_2_O_2_. However, in the case of an inorganic–organic hybrid without CuNPs, i.e., MCM and MCMCal exhibited ∼8% and ∼10% of the oxidation of TMB compared to that of Cu@MCMCal in the presence of H_2_O_2_. To further investigate the role of the calix[4]­arene in the nanoenzyme, a control experiment was carried out using copper NPs doped in MCM. The CuNPs and Cu@MCM showed ∼46% and ∼63% conversion of TMB to TMB_ox_ (Figure 3c). The corresponding spectra can be noticed from Supporting Information, SI09 and Figure S09a. However, Cu@MCMCal showed 150–200% higher activity toward peroxidase as compared to CuNPs and Cu@MCM. This suggests the presence of calix[4]­arene in the nanoenzyme allows the reactants to the reaction center by facilitating it through noncovalent interactions and thereby increasing the rate of reaction. Therefore, the synergistic effects of CuNPs, calix[4]­arene, and mesoporous silica make the nanoenzyme a robust and efficient catalyst toward peroxidase activity.

**Figure 3:**
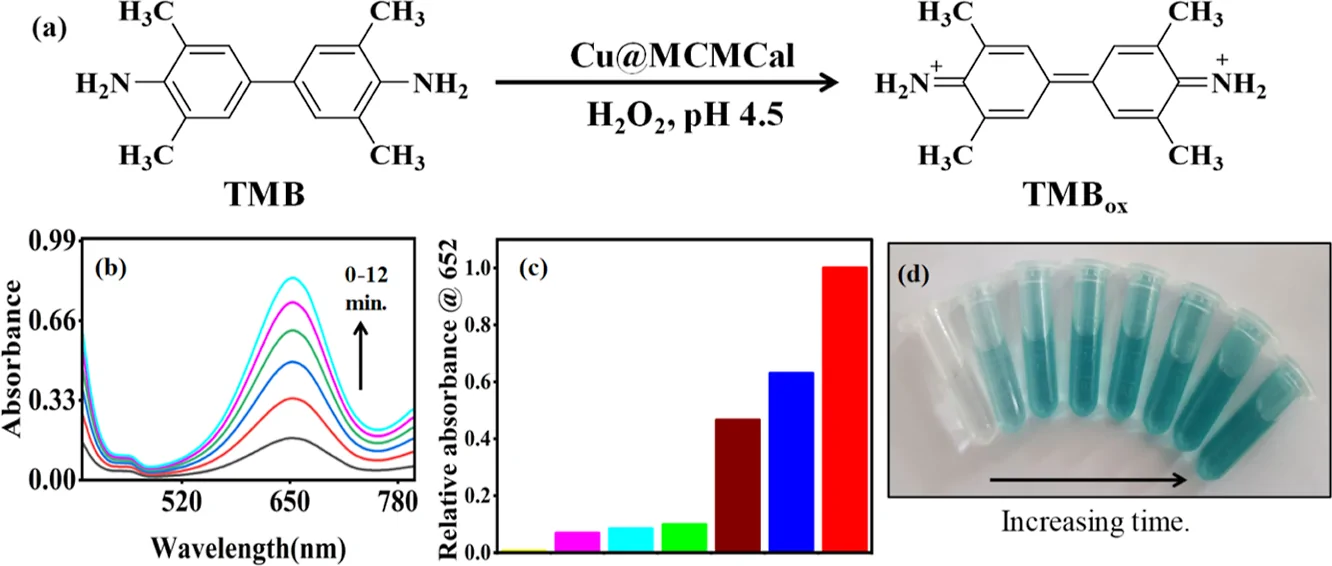
(a) Schematic measurement of Cu@MCMCal-catalyzed oxidation of TMB in the presence of H_2_O_2_ at pH 4.5; (b) time-dependent UV–visible absorption spectra recorded for 12 min where TMB—0.5 mM; H_2_O_2_—80.6 mM; and Cu@MCMCal—50 μg/mL; (c) comparison of peroxidase activity: Relative absorbance at λ_max_ = 652 nm for TMB oxidation in the presence of only catalyst (yellow, no oxidant); only H_2_O_2_ (magenta, no catalyst); MCM + H_2_O_2_ (cyan); MCMCal + H_2_O_2_ (green); CuNPs + H_2_O_2_ (wine); Cu@MCM + H_2_O_2_ (blue); and Cu@MCMCal + H_2_O_2_ (red) using TMB (0.5 mM),H_2_O_2_ (30 mM), and Cu@MCMCal—50 μg/mL; and (d) photograph showing increase in peroxidase activity (increase in blue color intensity) at different time intervals.

**Figure 4:**
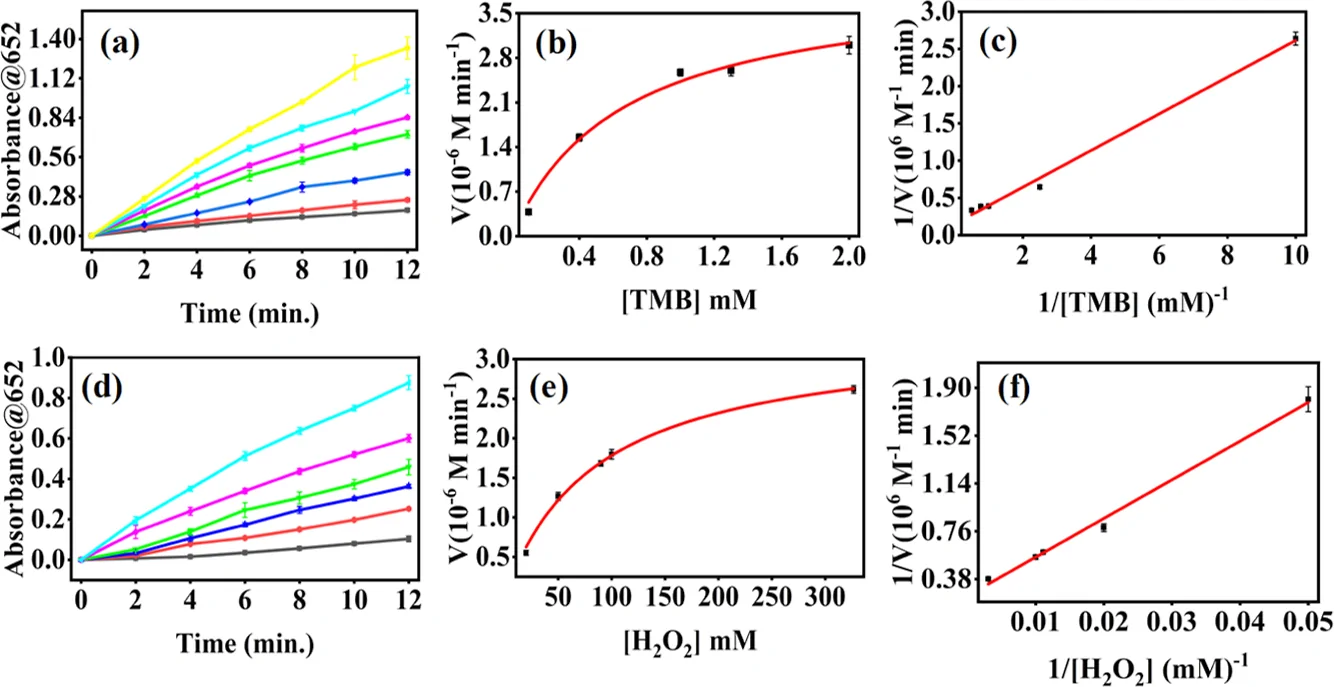
(a) Plot of absorbance at λ_max_ = 652 nm for TMB_ox_ recorded over 12 min by using the conc. Of TMB as 0.1, 0.2, 0.3, 0.4, 0.5, 0.6, and 0.7 mM,H_2_O_2_ as 25 μL of 30% w/w; (b) Michaelis–Menten plot having initial velocity vs concentration of TMB; (c) Lineweaver–Burk plot (1/*V* (10^6^ M^–1^ min) vs [TMB]^−1^ (mM)^−1^; (d) time-dependent plot of absorbance plot at λ_max_ = 652 nm by varying concentrations of H_2_O_2_ as 10, 20, 30, 40, 50, and 80.6 mM over 12 min and TMB-0.5 mM; and plots (e,f) are Michaelis–Menten and Lineweaver–Burk plots, for variable concentrations of H_2_O_2_, respectively. In all the measurements, conc. of Cu@MCMCal is 50 μg/mL.

The kinetic parameters of oxidation of TMB in the presence of H_2_O_2_ were evaluated using Michaelis–Menten and Lineweaver–Burk models. The TMB concentration was varied from 0.1 to 2 mM and time-dependent absorption spectra were recorded (SI04, Figure S04). The absorption at λ_max_ = 652 nm vs time has been plotted for all the concentrations (0.1, 0.2, 0.3, 0.4, 0.5, 0.6, and 0.7 mM) of TMB as can be depicted from Figure 4a. The linear regression of the initial time-dependent absorption plots of oxidation of TMB up to 12 min was used to calculate the initial velocities. The molar extinction coefficient (ε) for TMB_ox_ is 39,000 M^–1^ cm^–1^. The Michaelis–Menten saturation curve was obtained by plotting a change in the initial velocities with varying TMB concentrations as shown in Figure 4b. The reciprocal plot of the Michaelis–Menten curve produced a linear Lineweaver–Burk line (Figure 4c), further supporting the accuracy of kinetic data. The maximum velocity (*V*_max_) and Michaelis–Menten constant (*K*_m_) of Cu@MCMCal toward peroxidase activity by varying the TMB concentration using the Michaelis–Menten plot obtained are *V*_max_ = 4.038 × 10^–6^ M min^–1^ and *K*_m_ = 0.661 ± 0.143 mM, respectively. The higher value of *V*_max_ suggests that the nanoenzyme saturates at a very high TMB concentration, which is further supported by the low *K*_m_ value suggesting the high catalytic affinity. From the ICP–MS analysis, the molar concentration of Cu [E] in Cu@MCMCal was found to be 5.84 × 10^–5^ M. The turnover number (*K*_cat_) calculated by (*V*_max_/[*E*]) and the corresponding catalytic efficiency (*K*_cat_/*K*_m_) were found to be 0.0692 min^–1^ and 1.05 × 10^2^ M^–1^ min^–1^, respectively. The specific activity of the catalyst is the conversion of TMB per minute by 1 mg of catalyst was calculated to be 0.08076 U mg^–1^

A similar experiment was performed by varying the H_2_O_2_ concentration from 10 to ∼300 mM by keeping TMB concentration constant as 0.5 mM and the nanoenzyme concentration as 50 μg/mL. Time-dependent absorption spectra and the plot of absorption vs time can be observed from Figure S05 and 4d. The *V*_max_ and *K*_m_ obtained from the Michaelis–Menten plot were found to be 3.321 × 10^–6^ M min^–1^ and 86.386 ± 6.844 mM, respectively. The increase in the value of *K*_m_ suggests the lower catalyst affinity at higher concentrations of H_2_O_2_ as compared to the higher TMB concentrations. The turnover number (*K*_cat_) and the corresponding catalytic efficiency (*K*_cat_/*K*_m_) were found to be 0.0569 min^–1^ and 0.659 × 10^2^ M^–1^ min^–1^, respectively.

The catalytic efficiency of the Cu@MCMCal nanoenzyme has been compared with the literature reported catalysts and enzymes. Table 1 represents a comparative data of the catalytic efficiency of the Cu@MCMCal nanoenzyme with the previously reported catalysts with respect to the *K*_m_ (Michaelis constant), *V*_max_, catalyst loading, catalyst efficiency, pH, reaction time, and recyclability toward peroxidase activity for the substrate TMB. Lowering values of *K*_m_ indicated a higher substrate affinity and excellent enzyme-mimicking activity. The synthesized Cu@MCMCal exhibits a low *K*_m_ for TMB (0.661 ± 0.143 mM) demonstrating strong substrate affinity compared to many previous literature reports, validating it as a potential candidate as high-performance nanoenzymes.

**Table 1:** Comparison of Cu@MCMCal with Literature Reported Catalysts as a Peroxidase Mimic Enzyme for TMB Substrate

nanoenzyme	*K*_m_ (mM)	*V*_max_ (M min^–1^)	catalyst loading	catalyst efficiency (M^–1^ min^–1^)	pH	reaction time	recyclability	refs
Cu/Nc	0.0786	1.806	2.5 μg/mL	-	4.0	10 min	-	^38^
Au-iron oxide nanocomposite (Fe3)	0.32	1.867 × 10^–7^	4 μg/mL	-	4.0	30 min	-	^39^
Zn-SA/CNClSAzyme	1.01	7.27 × 10^–4^	5 μg/mL	8.89 × 10^5^	4.0	10 min	-	^40^
CuN3-SAzyme	1.61	-	20 μg/mL	6.56 × 10^3^	3.54	-	-	^41^
etched Cu nanocubes	0.39	6.17 × 10^–7^	0.5 cm^2^ film/500 μL	-	3.0	15 min	16 cycles over 16 weeks	^42^
1.0 Cu-mC_3_N_6_	-	-	10 μg/mL	-	5.4	20 min	-	^43^
Ce-MOF	4.64	4.014 × 10^–4^	6.25 μg/mL	-	4.0	30 min	-	^44^
Cu_4_Co_6_ ZIF	0.022	-	0.1 mg/mL	-	4.0	15 min	-	^45^
Cu–N/O	1.24	1.314 × 10^–4^	8.4 μg/mL	1.152 × 10^5^	4.0	-	-	^46^
Cu-NC	0.10	2.33 × 10^–6^	6.3 μg/mL	-	4.5	30 min	-	^47^
Cu_2_O/hemin-GO	-	-	-	-	4.5	90 min	5 cycles	^48^
Cu_2_O@ASP	0.153	1.86 × 10^–4^	40 μg/mL	-	5.0	1 min 60 s	-	^49^
CuMCMCal	0.661 ± 0.143	4.038 × 10^–6^	50 μg/mL	1.05 × 10^2^	4.5	12 min	6 cycles	this work

The peroxidase mimic activity of Cu@MCMCal nanoenzyme was also explored toward the oxidation of another substrate OPD using H_2_O_2_ as an oxidant in PBS buffer (10 mM, pH 7.4) (Figure 5a). OPD has a tendency to undergo oxidation to 2,3-diaminophenazine (DAP) in the presence of an oxidant^50,51^ and the oxidation of OPD to DAP confirmed by a prominent broad absorption peak at λ_max_ = 417 nm. The absorption spectra were recorded with a gradual increase in time in the presence of H_2_O_2_ (50 mM) by nanoenzyme Cu@MCMCal (50 μg/mL) as can be observed from Figure 5b. An increase in the absorption peak intensity was observed with increasing time suggesting the oxidation of OPD to DAP. The formation of DAP was accompanied by distinct color changes from colorless to yellow can be noticed from the photograph in Figure 5c.

**Figure 5:**
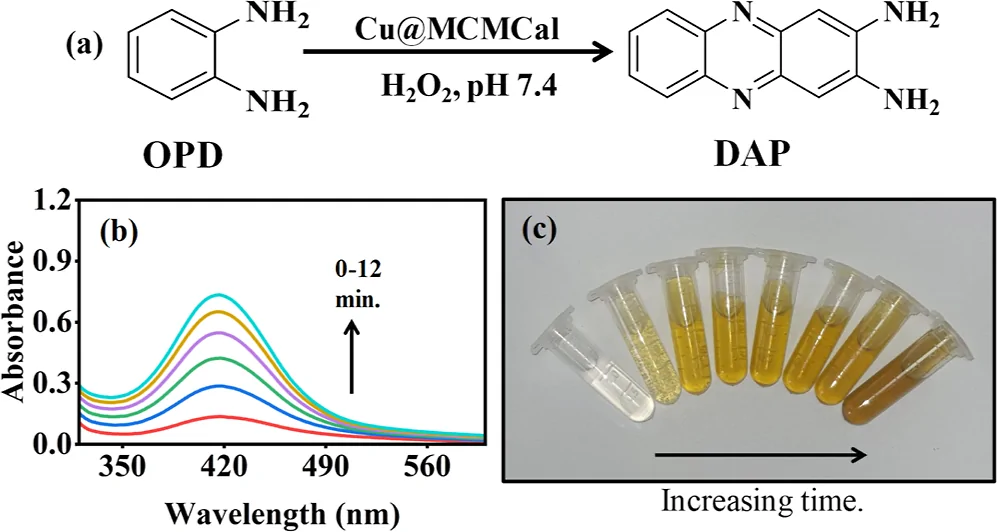
(a) Schematic representation of oxidation of OPD by using Cu@MCMCal nanoenzymes in the presence of H_2_O_2_ at pH 7.4; (b) the absorption spectra recorded up to 12 min at OPD concentration as 0.5 mM,H_2_O_2_ as 50 mM, and Cu@MCMCal as 50 μg/mL; and (c) photograph showing the increase in the yellow color intensity of DAP with increasing time.

To further calculate the kinetic parameters of Cu@MCMCal nanoenzyme, Michaelis–Menten and Lineweaver–Burk models were used (eqs 1 and 2). For the initial kinetic studies, the concentration of OPD was varied from 0.1 to 4 mM, and corresponding absorption spectra for the conversion of OPD to DAP were recorded over 12 min as given in SI06, Figure S06. The absorption of DAP at λ_max_ = 417 nm was plotted against time for different concentrations of OPD (0.1, 0.3, 0.4, 0.5, 0.8, 1.2, and 1.5 mM) as could be noticed from Figure 6a. The molar extinction coefficient (ε) for DAP is 16,700 M^–1^ cm^–1^. The calculated velocities from linear regression were plotted against varying concentrations of OPD as shown in Figure 6b. The Lineweaver–Burk plot was also obtained by plotting the reciprocal of initial velocity against the reciprocal of OPD concentration, as shown in Figure 6c. Based on the above measurements, *V*_max_ and *K*_m_ were calculated from the Michaelis–Menten plot and found to be 8.527 × 10^–6^ M min^–1^ and 0.606 ± 0.061 mM, respectively. The turnover number (*K*_cat_) was calculated by using the equation (*V*_max_/[*E*]) as 0.1458 min^–1^ and the corresponding catalytic efficiency (*K*_cat_/*K*_m_) was found to be 2.406 × 10^2^ M^–1^ min^–1^. The specific activity of the catalyst, which is the conversion of OPD per minute by 1 mg of catalyst, was calculated as 0.17054 U mg^–1^.

**Figure 6:**
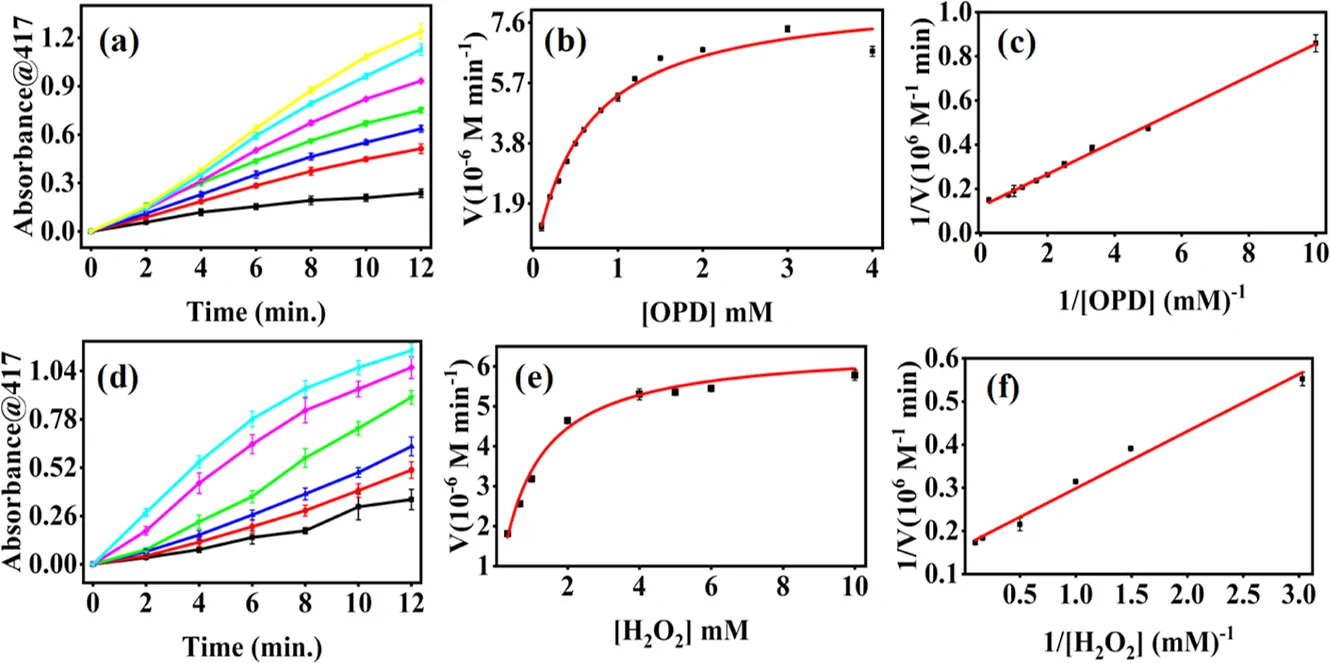
(a) Time-dependent absorbance recorded at λ_max_ = 417 nm for DAP formation over 12 min to evaluate the peroxidase-mimic (Cu@MCMCal—50 μg/mL, OPD—0.1 to1.5 mM, and H_2_O_2_—50 mM); (b) Michaelis–Menten plot of initial velocity (*V*(10^–6^ M min^–1^)) against the OPD concentration); (c) Lineweaver–Burk plot (1/*V* (10^6^ M^–1^ min)) vs [OPD]^−1^ (mM)^−1^ to calculate *K*_m_ and *V*_max_; (d) time-dependent absorbance recorded at λ_max_ = 417 nm for DAP formation over 12 min at varying H_2_O_2_ concentrations (0.33–10 mM) (Cu@MCMCal—50 μg/mL, and OPD—0.5 mM); and plots (e,f) are similar to (b,c) except the concentration of H_2_O_2_.

The studies were also carried out using H_2_O_2_ varied from 0.33 to 10 mM seems to affect OPD oxidation (Figure 6d and SI07, Figure S07) as similar to the case of TMB oxidation. The Michaelis–Menten and Lineweaver–Burk plots can be noticed from Figure 6e,f. By varying the conc. of H_2_O_2_, the values of *V*_max_ and *K*_m_ were found to be 6.486 × 10^–6^ M min^–1^ and 0.910 ± 0.102 mM, respectively. The turnover number (*K*_cat_) and the corresponding catalytic efficiency (*K*_cat_/*K*_m_) were found to be 0.1110 min^–1^ and 1.219 × 10^2^ M^–1^ min^–1^, respectively.

Overall, the substrate scope was evaluated for Cu@MCMCal toward peroxidase activity using TMB and OPD. Ongoing from TMB to OPD, the value of *V*_max_ was increased by 2.1 times and the *K*_m_ value was lowered, suggesting the better affinity of the Cu@MCMCal nanoenzyme toward the OPD.

The catalytic efficiency of the Cu@MCMCal nanoenzyme has been compared with the literature reported catalysts and enzymes. Table 2 represents a comparative data of the catalytic efficiency of the Cu@MCMCal nanoenzyme with the previously reported catalysts for the substrate OPD. The synthesized Cu@MCMCal exhibits a low *K*_m_ for OPD (0.606 ± 0.061 mM) demonstrating a strong substrate affinity compared to many previously reported nanoenzymes.

**Table 2:** Comparison of Cu@MCMCal with Literature Reported Catalysts as a Peroxidase Mimic Enzyme for OPD Substrate

nanoenzyme	*K*_m_ (mM)	*V*_max_ (M min^–1^)	catalyst loading	catalyst efficiency (M^–1^ min^–1^)	pH	reaction time	recyclability	refs
2D Co-MOF	1.87	2.18 × 10^–4^	41.15 μg/mL	-	4.5	10 min	-	^52^
CTF-NPs	0.819	1.62 × 10^–6^	1 mg/mL	-	-	10 min	-	^53^
MnFe_2_O_4_	27.5 ± 2.6	6.24 × 10^–6^	60 μg/mL	-	7.0 ± 0.1	30 min	-	^54^
CoFe_2_O_4_	32.7 ± 1.9	4.68 × 10^–6^	60 μg/mL	-	7.0 ± 0.1	30 min	-	^54^
NiFe_2_O_4_	8.4 ± 1.0	5.16 × 10^–7^	60 μg/mL	-	7.0 ± 0.1	30 min	-	^54^
ZnFe_2_O_4_	22.6 ± 2.4	4.92 × 10^–7^	60 μg/mL	-	7.0 ± 0.1	30 min	-	^54^
BSA-MnO_2_ NPs	0.31	4.926 × 10^–6^	3.7 μg/mL	163.68	4.0	5 min	-	^55^
Cu3/ND@G	2.89	6.9 × 10^–6^	0.1 mg/mL	-	4.5	10 min	-	^56^
0.4Cu-CDs + Fe^2+^	0.644	4.961 × 10^–8^	50 μg/mL	-	7.0	25 min	-	^57^
PMOF(Fe)	7.81	-	25 μg/mL	-	7.4	-	-	^58^
CuMCMCal	0.606 ± 0.061	8.527 × 10^–6^	50 μg/mL	2.406 × 10^2^	7.4	12 min	6 cycles	this work

The order of the peroxidase-mimic reaction of Cu@MCMCal nanoenzyme with respect to TMB was determined by plotting the natural logarithm of the initial reaction rate against the natural logarithm of TMB concentration. For this purpose, the increase in absorbance at λ_max_ = 652 nm was monitored over time at different concentrations of TMB (0.4, 0.5, 0.6, and 0.7 mM) (Figure 7a). The initial reaction rates were calculated from the absorbance–time plots and obtained rates were used to plot ln­(rate) vs ln­[TMB]. The plot of ln­(rate) vs ln­[TMB] fitted to a linear line having a slope of 1.13 (*R* ^2^ = 0.98), indicating that the peroxidase reaction is a first order with respect to TMB (Figure 7b).

**Figure 7:**
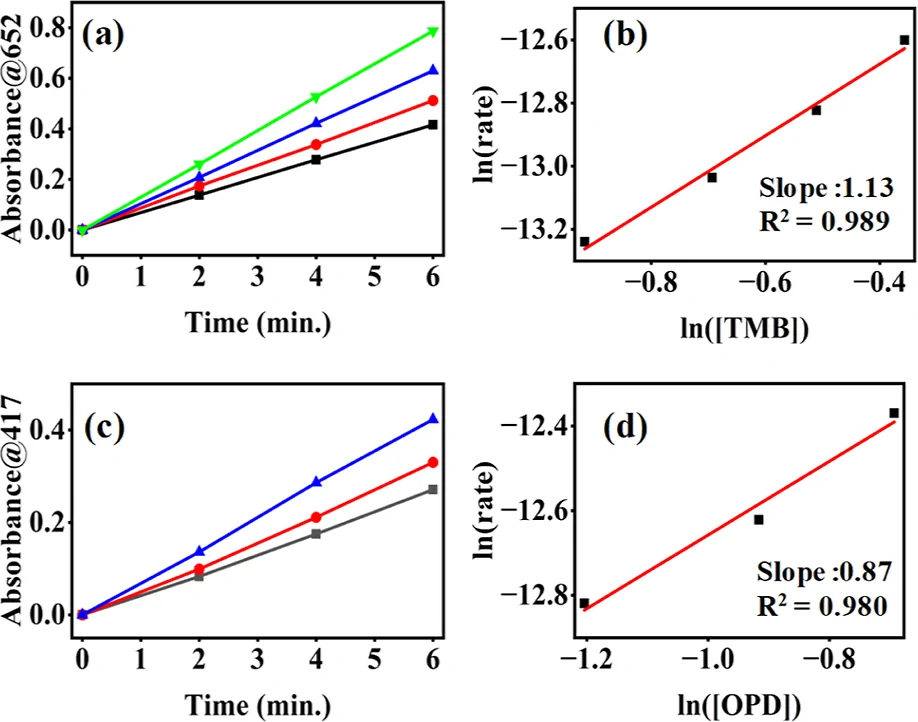
(a,c) Plots of absorbance at λ_max_ = 652 for TMB and at λ_max_ = 417 nm for OPD recorded up to 6 min using TMB conc. (0.4, 0.5, 0.6, and 0.7 mM) and OPD conc. (0.3, 0.4, and 0.5 mM). In both the cases, Cu@MCMCal was 50 μg/mL, H_2_O_2_ was 25 μL of 30% w/w, and 50 mM for TMB and OPD and (b,d) graphs between ln­(rate) vs ln­(substrate) for TMB and OPD, respectively.

Similarly, the order of oxidation of OPD was calculated by recording the absorbance spectra at λ_max_ = 417 nm with respect to time at different (0.3, 0.4, and 0.5 mM) OPD concentrations (Figure 7c). The initial reaction rates obtained from these plots were used to construct a graph of ln­(rate) vs ln­[OPD]. The slope of the straight line fit was found to be 0.87 (*R* ^2^ = 0.98), which is close to unity (Figure 7d). These results suggest that the oxidation of both the substrate TMB and OPD in the presence of H_2_O_2_ using the Cu@MCMCal nanoenzyme follows first-order kinetics.

### Oxidase-Like Activity

The oxidase-like activity of Cu@MCMCal nanoenzyme was evaluated by studying the oxidation of dopamine in PBS buffer (10 mM, pH 7.4). The oxidation of dopamine was confirmed by the appearance of a broad peak at λ_max_ = 480 nm (Figure 8a,b). This absorption corresponds to the formation of aminochrome/dopaminechrome-type oxidation products reported during dopamine oxidation.^59–61^ The absorption titration was performed with respect to time over a period of 50 min.^61^ The oxidation of dopamine to aminochrome/dopaminechrome type can also be noticed from the color change from colorless to light pink as can be noticed from the inset of Figure 8b. The plot of absorbance at λ_max_ = 480 nm vs time in the presence and absence of the nanoenzyme has been compared (Figure 8c). The data suggested that the oxidation of dopamine in the presence of Cu@MCMCal was approximately six times higher than that observed without the Cu@MCMCal nanoenzyme (Figure 8d). Therefore, Cu@MCMCal has also been found as a potential oxidase mimic.

**Figure 8:**
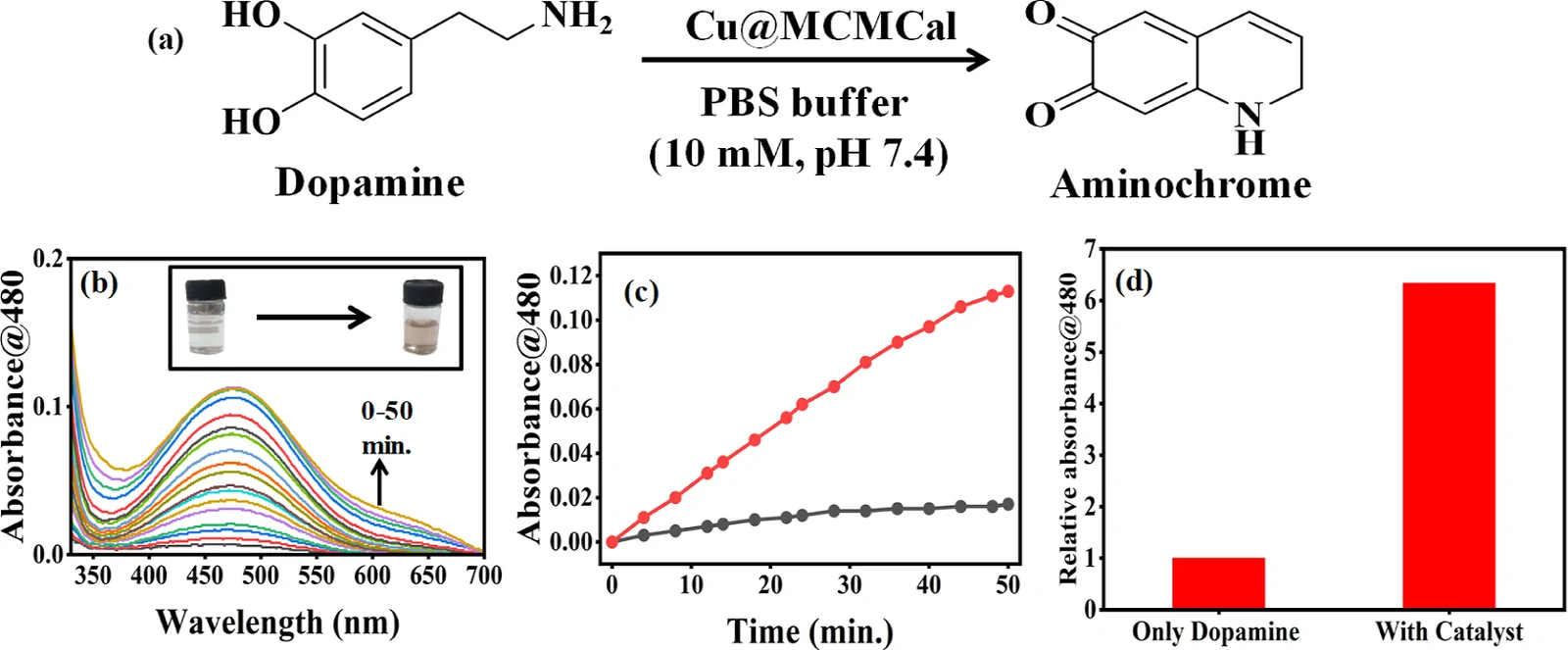
(a) The schematic representation of oxidation of dopamine to aminochrome/dopaminechrome-type; (b) UV–visible absorption spectra recorded with increasing time toward the oxidation of dopamine (80 μM) in the presence of Cu@MCMCal (50 μg/mL) and the inset shows the vials depicting the color change on going from the dopamine to aminochrome/dopaminechrome type; (c) the plot of absorption at λ_max_ = 480 nm vs time in minutes showing the comparison of oxidation of dopamine in the absence (black) and presence (red) of Cu@MCMCal nanoenzymes; and (d) the comparison of relative absorption intensity at λ_max_ = 480 nm in the presence and absence of Cu@MCMCal.

To check the robustness of nanoenzyme, Cu@MCMCal, the recyclability studies were carried out using the TMB substrate. The absorption peak at λ_max_ = 652 nm was monitored with respect to time in each cycle. The inorganic–organic hybrid nanoenzyme, i.e., Cu@MCMCal, retained 98% of its initial catalytic activity after the first cycle. The recyclability studied performed up to six cycles. The catalytic activity decreased by ∼25% up to the sixth cycle during recyclability (Figure 9a). The recycled nanoenzyme further analyzed by PXRD and XPS exhibits all the peaks corresponding to the Cu@MCMCal suggesting the stability of the nanoenzyme (SI08, Figure S08a,b). Many of the catalysts had limited recyclability, however, our hybrid material (Cu@MCMCal) recycled up to six times showed only one-fourth of the loss in catalytic efficiency.^59^ The ICP–MS analysis of recycled catalyst showed that Cu content in recycled Cu@MCMCal was 5.50 wt % as compared to the Cu content of 7.43 wt % in fresh Cu@MCMCal. There is approximately 1.92 wt % of Cu leached out during the recycled process until six cycles. This suggests the Cu-doped MCMCal material, i.e., Cu@MCMCal, is a highly robust nanoenzyme toward the oxidation of organic substrates, i.e., TMB. The minor loss in catalytic reactivity could be due to the blockage of active sites by reaction intermediates and byproducts or Cu leaching during the peroxidase-mimic activity.

**Figure 9:**
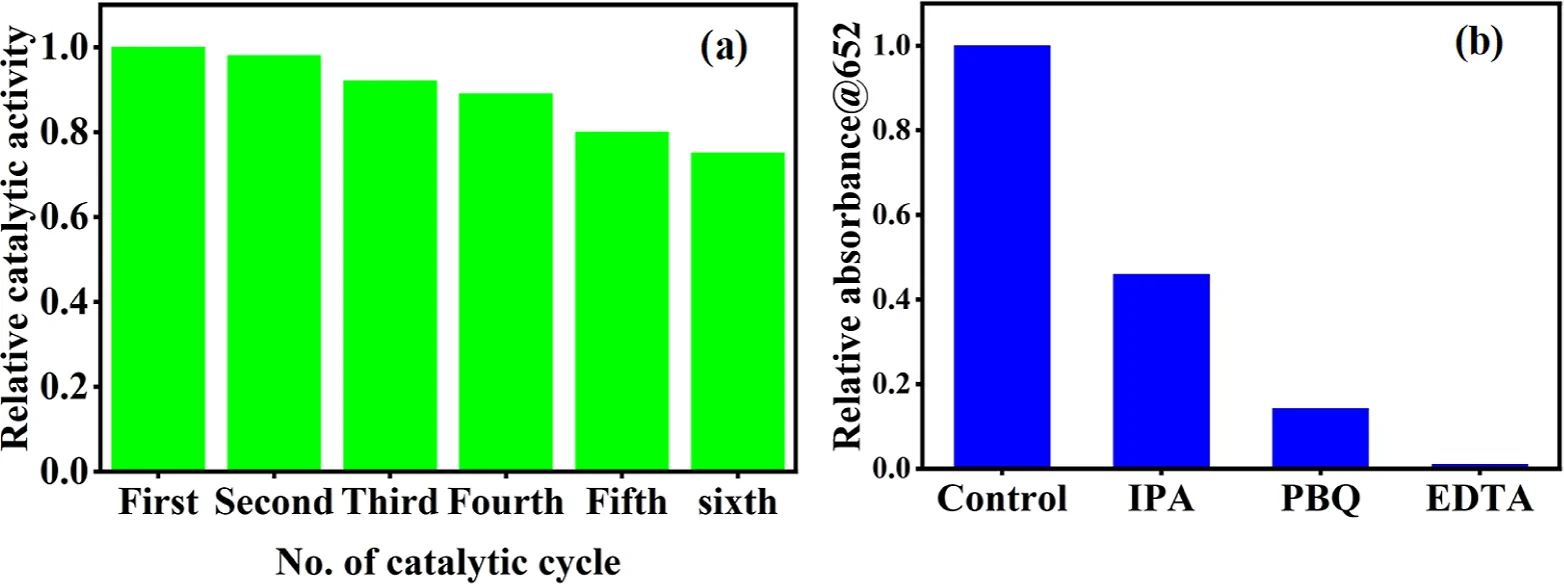
(a) A bar diagram illustrating the relative catalytic activity of the Cu@MCMCal nanoenzyme over six successive catalytic cycles and (b) bar graph showing the relative absorbance at 652 nm in the presence of different scavenger agents, i.e., IPA, PBQ, and EDTA.

A scavenging experiment was performed to identify the reactive oxygen species (ROS) involved in the oxidation of TMB catalyzed by Cu@MCMCal nanoenzymes in the presence of H_2_O_2_. Isopropanol (IPA) was employed as a scavenger for hydroxyl radicals (^•^OH), *p*-benzoquinone (PBQ) used to quench the superoxide radicals (O_2_^•–^), and EDTA worked as an inhibitor for the catalytic activity of Cu ions along with the evaluation of the role of Fenton-like mechanism. In the case of IPA, the absorption intensity was decreased to 46.1% (quenching of TMB oxidation reaction was ∼54%) as compared to the control (oxidation of TMB in the presence of Cu@MCMCal and H_2_O_2_), indicating the formation of hydroxyl radicals. Upon addition of PBQ, the absorption intensity decreased to ∼14% (quenching of TMB oxidation reaction was ∼86%), indicating the strong involvement of superoxide radicals (O_2_^•–^). However, in the presence of EDTA, no oxidation of TMB to TMB_ox_ was observed, suggesting the complexation of Cu ions with EDTA (Figure 9b), indicating that the metal ions play an essential and significant role in the catalysis, and the reaction proceeded through the Fenton-like mechanism. The corresponding absorption spectra are given in the Supporting Information, SI09 and Figure S09b. Therefore, the scavenging results and the proposed Cu redox cycling support that the reaction follows the Fenton-like pathway as a plausible mechanism involving ^•^OH and O_2_^•–^ radicals during the peroxidase-mimic activity of Cu@MCMCal. This data suggests that involvement of O_2_^•–^ radical ∼3.4 fold higher than the ^•^OH radical.

Initially, the reaction proceeds via the adsorption of TMB molecules on its nanoenzyme surface through the noncovalent interactions within the porous calixarene-MCM framework. Upon addition of H_2_O_2_, the Cu^+^ species present on the catalyst surface interact with H_2_O_2_ to form an unstable copper–peroxo intermediate (step 1). This intermediate decomposes rapidly and undergoes the oxidation of Cu^+^ to Cu^2+^ along with the formation of hydroxyl radicals (^•^OH) (step 2). Simultaneously, metallic Cu^0^ NPs on the nanoenzyme surface undergo oxidation reaction by releasing an electron to generate catalytically active Cu^+^ species (step 3). The generated Cu^+^ can further react with dissolved oxygen in acetate buffer to produce superoxide radicals (O_2_^•–^) along with Cu^2+^ (step 4). However, O_2_^•–^ radicals can also be formed via the reaction of ^•^OH radicals with H_2_O_2_ (step 5). The regeneration of the nanoenzyme was achieved by the reduction of Cu^2+^ back to Cu^+^ by O_2_^•–^ radicals and surface electrons as can be noticed from (steps 6 and 7).

$$Step\;1:\;Cu^{+}+HO-OH\to \underset{copper‐peroxo\;intermediate}{\left[Cu^{2+}\cdot \cdot \cdot OH\cdot \cdot \cdot OH\right]}$$

Step2:[Cu2+···OH···OH]→Cu2++O·H+−OH

$$Step\;3:\;{Cu}^{0}{\to Cu}^{+}+e^{-}$$

$$Step\;4:\;{Cu}^{+}+O_{2}{\to Cu}^{2+}+{O_{2}}^{\cdot -}$$

$$Step\;5:\cdot \;OH+HO-OH\to {O_{2}}^{\cdot -}+H^{+}+H_{2}O$$

$$\mathrm{Step}\;6:\;{Cu}^{2+}+{O_{2}}^{\cdot -}\to {Cu}^{+}+O_{2}$$

$$Step\;7:\;{Cu}^{2+}+surface\;e^{-}\to {Cu}^{+}$$

The generated reactive oxygen species, mainly ^•^OH and O_2_^•–^, oxidized TMB into a blue-colored product (TMB_ox_). Finally, the oxidized TMB molecule undergoes desorption from the catalyst surface leaving active sites available for the next catalytic cycle (Figure 10). Overall, Cu@MCMCal behaves as a peroxidase enzyme mimic via hydroxyl (^•^OH) and (O_2_^•–^) superoxide radicals.

**Figure 10:**
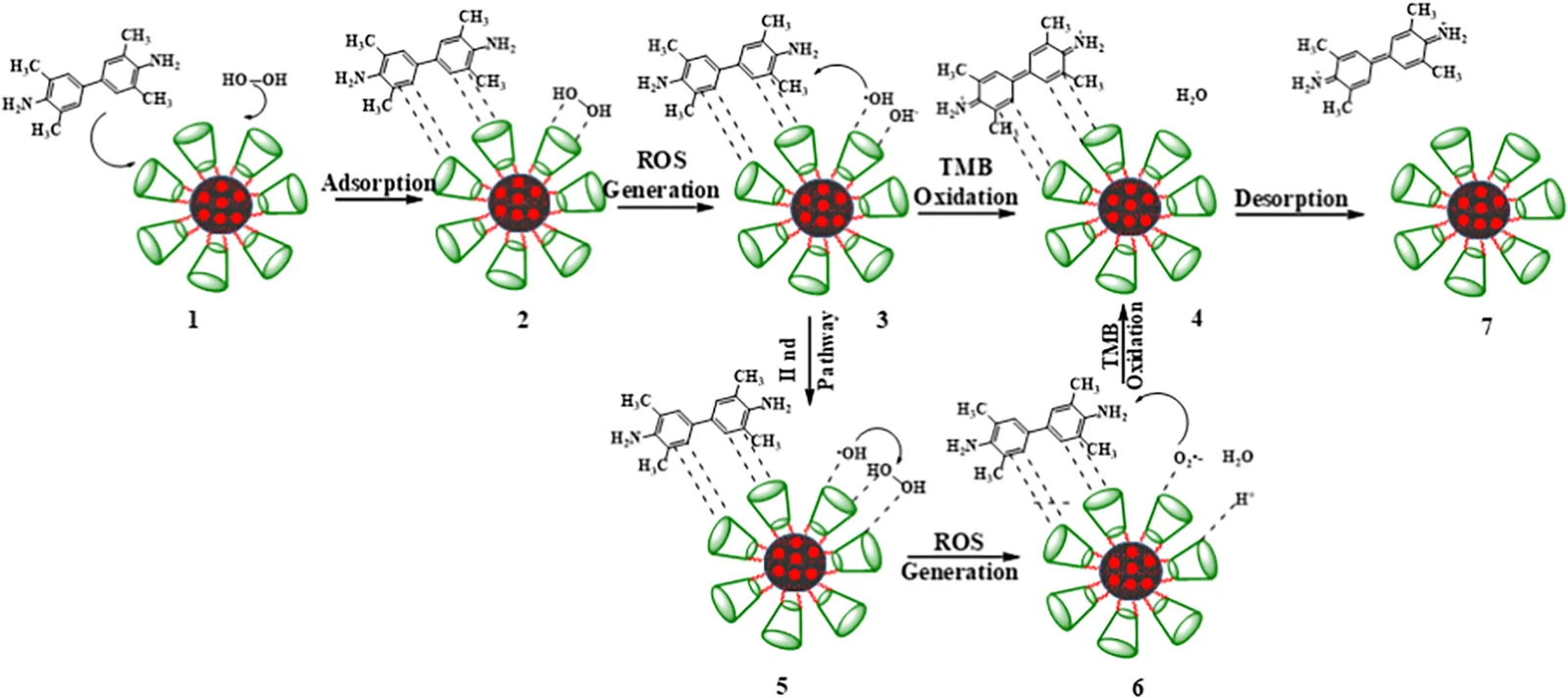
Proposed mechanism for peroxidase-like activity for Cu@MCMCal nanoenzymes.

## Conclusion

A inorganic–organic hybrid nanoenzyme, i.e., Cu@MCMCal, has been designed and synthesized. Initially, mesoporous silica (MCM-41) was covalently functionalized with calix[4]­arene through its lower rim using GPTMS, resulting in the formation of the MCMCal hybrid nanomaterial. CuNPs were synthesized by the chemical reduction method. These as-synthesized CuNPs were doped on to the surface of the MCMCal nanohybrid to form Cu@MCMCal nanomaterials. The successful immobilization of calix[4]­arene on the MCM-41 surface and doping of CuNps to it was confirmed by various spectroscopic and microscopic techniques. MCMCal exhibits all the peaks corresponding to MCM-41 suggesting the covalent functionalization of MCM-41 by calix[4]­arene based on the UV–Vis and FTIR measurements. TGA analysis revealed the amount of calix[4]­arene immobilized on MCM-41 was ∼13%. The oxidation state of copper in CuNps and Cu@MCMcal was analyzed by PXRD and XPS measurements. The results indicated that CuNPs are formed as Cu^0^ in bulk which oxidized to Cu^2+^ from the surface. After doping in the MCMCal nanohybrid, a shift in the oxidation state from Cu^2+^ to Cu^+^/Cu^0^ was observed, indicating the strong interaction between the copper species and calix[4]­arene moiety in the synthesized nanoenzyme. ICP–MS analysis further confirmed the copper loading in the Cu@MCMCal nanoenzyme to be 7.42 wt %. TEM analysis revealed the particle size of synthesized Cu@MCMCal nanoenzyme is ∼200–250 nm and has a porous morphology. The incorporation of CuNPS in the nanoenzyme was further confirmed by EDX-mapping and dark-field STEM imaging.

This Cu@MCMCal hybrid has been explored as a peroxidase mimic toward the oxidation of TMB and OPD substrates in the presence of H_2_O_2_ and the oxidation mimic toward the oxidation of dopamine. The kinetic parameters for the peroxidase mimic were determined by using the Michaelis–Menten model. For TMB as the substrate, the calculated *K*_m_ value was found to be 0.661 ± 0.143 mM. The corresponding *k*_cat_ and catalytic efficiency (*k*_cat_/*K*_m_) were calculated to be 0.0692 min^–1^ and 1.05 × 10^2^ M^–1^ min^–1^, respectively. In comparison, when the H_2_O_2_ concentration was varied, the corresponding *K*_m_ value was found to be 86.386 ± 6.844 mM. Similarly, for OPD, the *K*_m_ was found to be 0.606 ± 0.061 mM and the corresponding *k*_cat_ and catalytic efficiency (*k*_cat_/*K*_m_) were calculated to be 0.145 min^–1^ and 2.406 × 10^2^ M^–1^ min^–1^, respectively, while for H_2_O_2_, *K*_m_ was 0.910 ± 0.102 mM. The significantly lower *K*_m_ value for TMB and OPD indicates that the nanoenzyme exhibits a stronger affinity toward TMB and OPD than H_2_O_2_. This behavior can be explained due to the hydrophobic cavity of calix[4]­arene playing a significant role for the adsorption of substrate molecule on to the surface of nanoenzymes. The Cu@MCMCal nanoenzyme was also able to oxidize the dopamine to aminochrome/dopaminechrome-type, which represents the versatility of the nanoenzyme. To evaluate the catalytic mechanism, radical scavenging experiments were performed using IPA, PBQ, and EDTA, confirming the strong involvement of superoxide radical (O_2_^•–^) over a hydroxide radical (^•^OH) during the oxidation process. The stability and robustness of the nanoenzyme was demonstrated by performing the recyclability studies. The nanohybrid material, i.e., Cu@MCMCal, showed excellent catalytic stability and reusability up to six consecutive cycles without significant loss in the catalytic activity.

## Supplementary Material



## Notes

PXRD, FTIR and XPS spectra of CUNPs, XPS spectra of Cu@MCMCal, Cu LMM Auger spectra for Cu@MCMCal, time-dependent UV–visible absorption spectra for varying conc. of TMB/OPD keeping H_2_O_2_ constant, time-dependent UV–visible absorption spectra for varying conc. of H_2_O_2_ keeping TMB/OPD constant, PXRD and XPS of recycled Cu@MCMCal, scavenging experiment and low-angle PXRD (PDF)
