# Supplementary material for: Development of Supramolecular Calix[4]arene-MCM-Based Nanomaterials Doped with CuNPs: An Inorganic–Organic Hybrid Recyclable Nanoenzyme as a Potent Peroxidase and Oxidase Mimic
Source: ACS Omega. 2026 Oct 16;11(43):67147–59. doi: 10.1021/acsomega.6c07489 (PMC13737829; doi:10.1021/acsomega.6c07489)
Supplement: Supplementary file 1 [file ao6c07489_si_001.pdf]

Supporting Information

Development of Supramolecular Calix[4]arene-MCM based nanomaterial doped with CuNPs: An Inorganic Organic Hybrid Recyclable Nanoenzyme as a Potent Peroxidase and Oxidase Mimic

Meenakshi Goyal,[a] Anuj Kumar,[b] Pardeep Kumar*[c], Bhawna Uttam*[a]

Corresponding Author: gcb8up@virginia.edu, bhawnauttam@jcboseust.ac.in

a. **Department of Chemistry, J.C. Bose University of Science and Technology, YMCA, Faridabad, Haryana-121006, India**
b. **Department of Chemistry, Indian Institute of Technology Bombay, Powai, Mumbai, 400076, India**
c. **Department of Chemical Engineering, University of Virginia, Charlottesville, 22904, USA**

Table of Contents

SI01. PXRD, FTIR and XPS spectra of CUNPs.

SI02. XPS spectra of Cu@MCMCal.

SI03. BET plots of MCM-41 and MCMCal.

SI04. Time dependent UV–visible absorption spectra for varying conc. of TMB keeping H_2O_2 constant.

SI05. Time dependent UV–visible absorption spectra for varying conc. of H_2O_2 keeping TMB constant.

SI06. Time dependent UV–visible absorption spectra for varying conc. of OPD keeping H_2O_2 constant

SI07. Time dependent UV–visible absorption spectra for varying conc. of H_2O_2 keeping OPD constant.

SI08. PXRD and XPS of recycled Cu@MCMCal

SI09. UV-Vis absorption spectra for compare the peroxidase activity of different nanohybrid formed and scavenging experiment.

SI10. Low-angle PXRD of MCM-41, MCMCal and Cu@MCMCal.

SI01. PXRD, FTIR and XPS spectra of CUNPs.

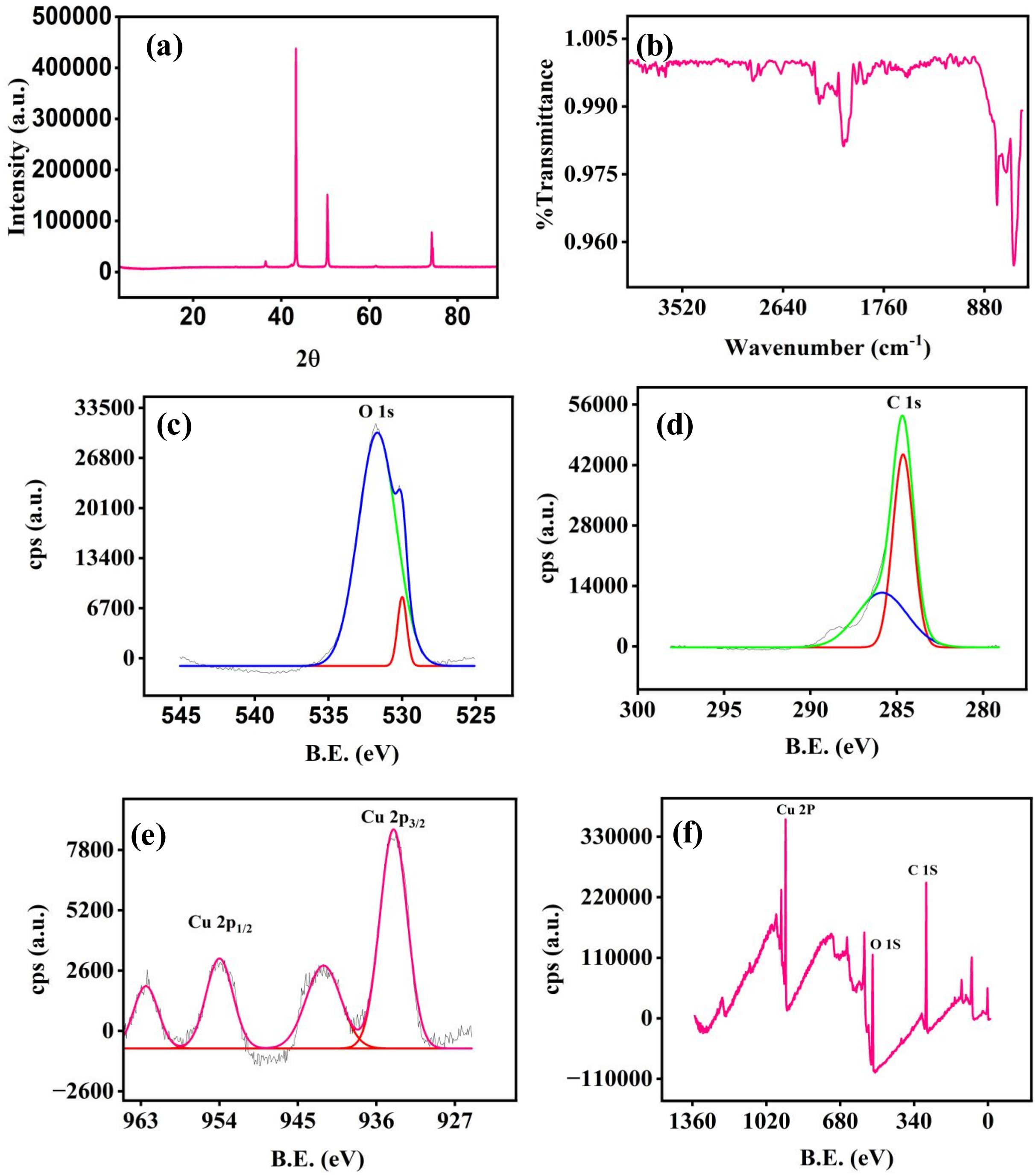

Figure S01. (a) PXRD; (b) FTIR and XPS spectral data for the Cu NPs (c) O; (d) C; (e) Cu and (f) survey xps spectrum

SI02. XPS data of Cu@MCMCal.

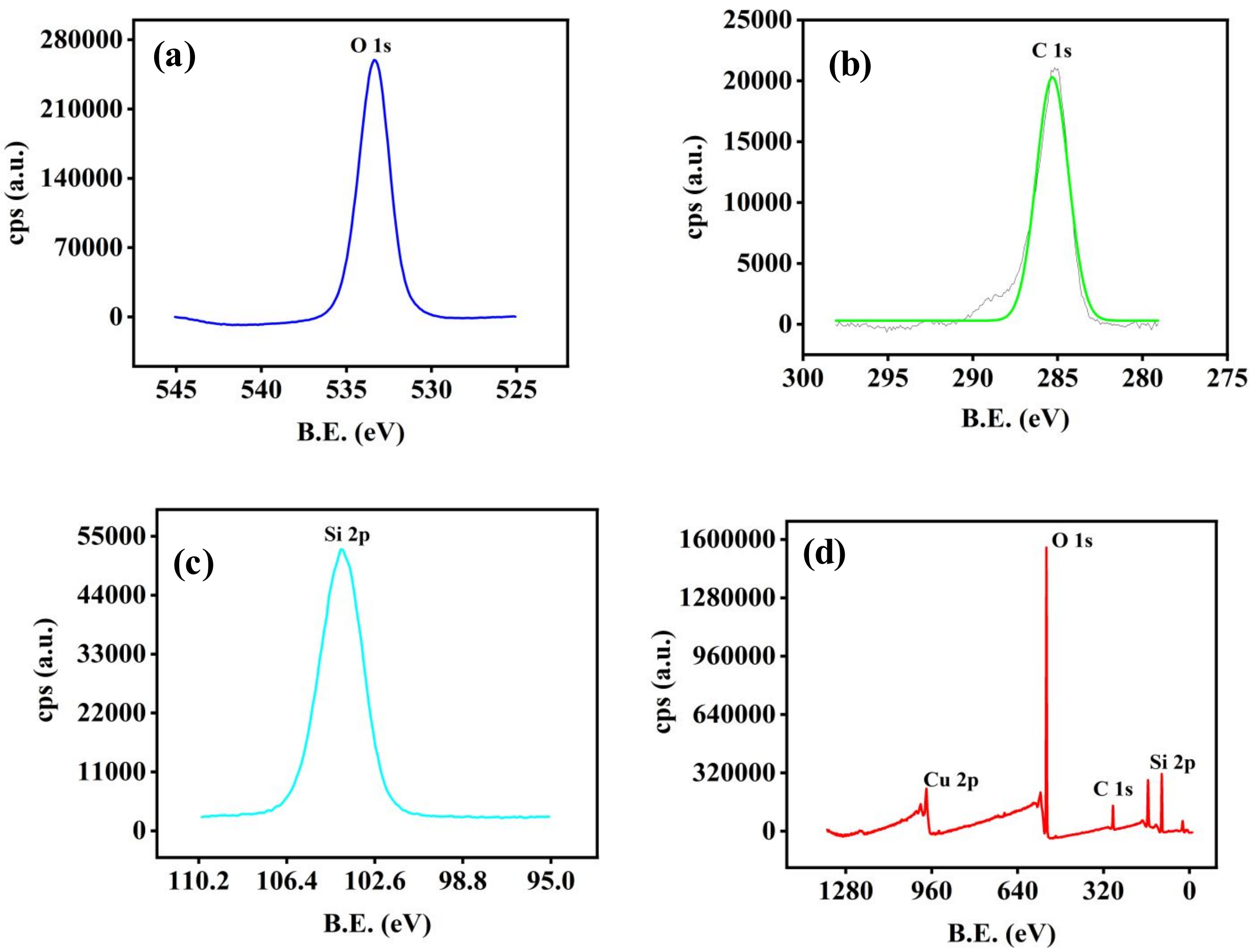

Figure S02. XPS spectral data for the Cu@MCCal (a) O; (b) C; (c) Si; and (d) survey xps spectrum.

SI03. Cu LMM Auger plots for Cu@MCMCal.

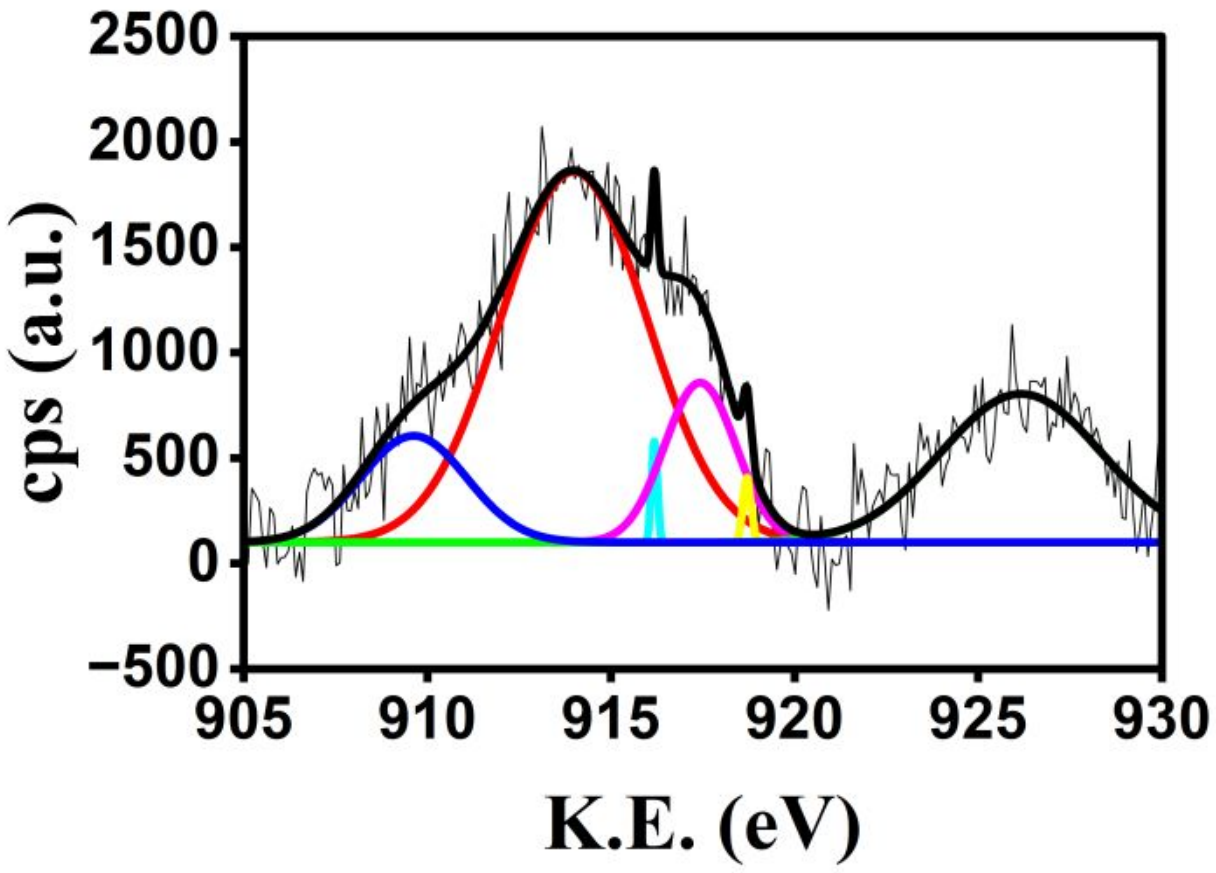

Figure S03. Cu LMM Auger plots for Cu@MCMCal.

SI04. Time dependent UV–visible absorption spectra for varying conc. of TMB keeping H_2O_2 constant.

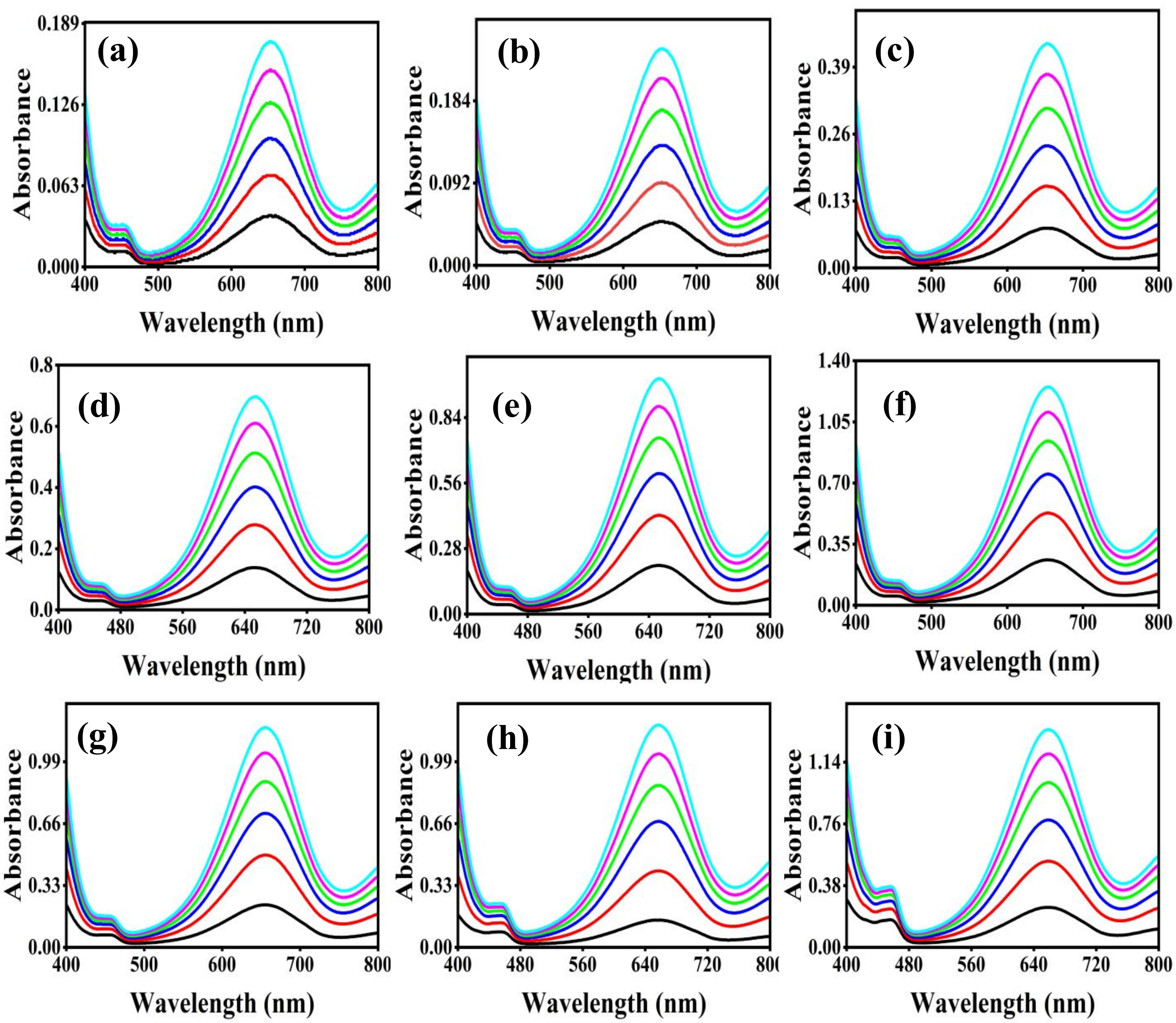

Figure S04. Time-dependent UV–visible absorption spectra recorded for 12 min. at an interval of 02 minutes at varying conc. Of TMB (a) 0.1mM (b) 0.2 mM (c) 0.3mM (d) 0.4 mM (e) 0.6mM (f) 0.7 mM (g) 1mM (h) 1.3 mM (i) 2mM by keeping constant conc. of H_2O_2- 25 μL of 30% w/w and Cu@MCMCal- 50 μg/mL .

SI05. Time dependent UV–visible absorption spectra for varying conc. of H_2O_2 keeping TMB constant.

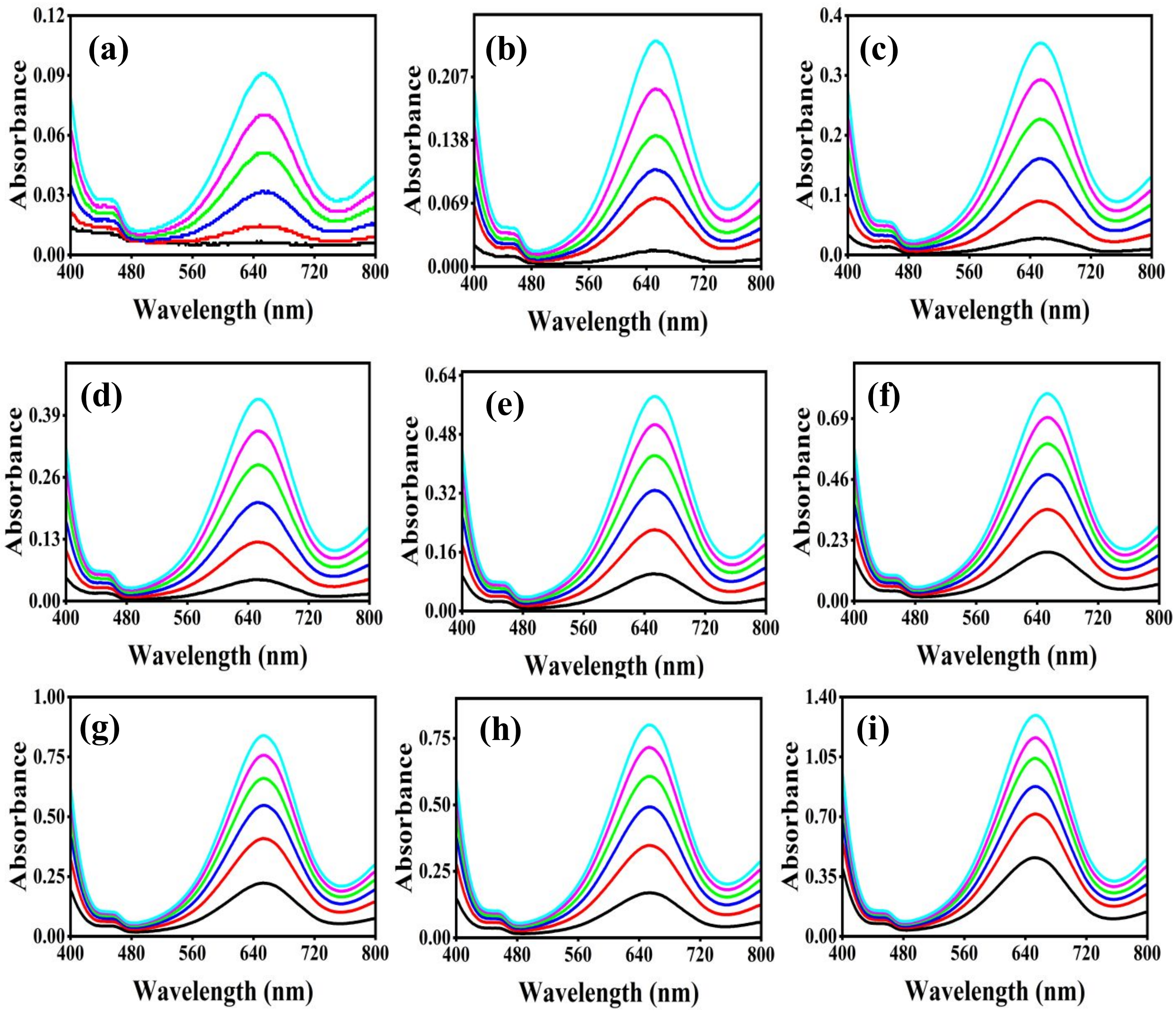

Figure S05. Time-dependent UV–visible absorption spectra recorded for 12 min. at an interval of 02 minutes at varying conc. of H_2O_2 (a) 10 mM (b) 20 mM (c) 30 mM (d) 40 mM (e) 50 mM (f) 80.6 mM (g) 100 mM (h) 110 mM (i) 326.6 mM by keeping constant conc. of TMB- 0.5 mM and Cu@MCMCal- 50 μg/mL .

SI06. Time dependent UV–visible absorption spectra for varying conc. of OPD keeping H_2O_2 constant

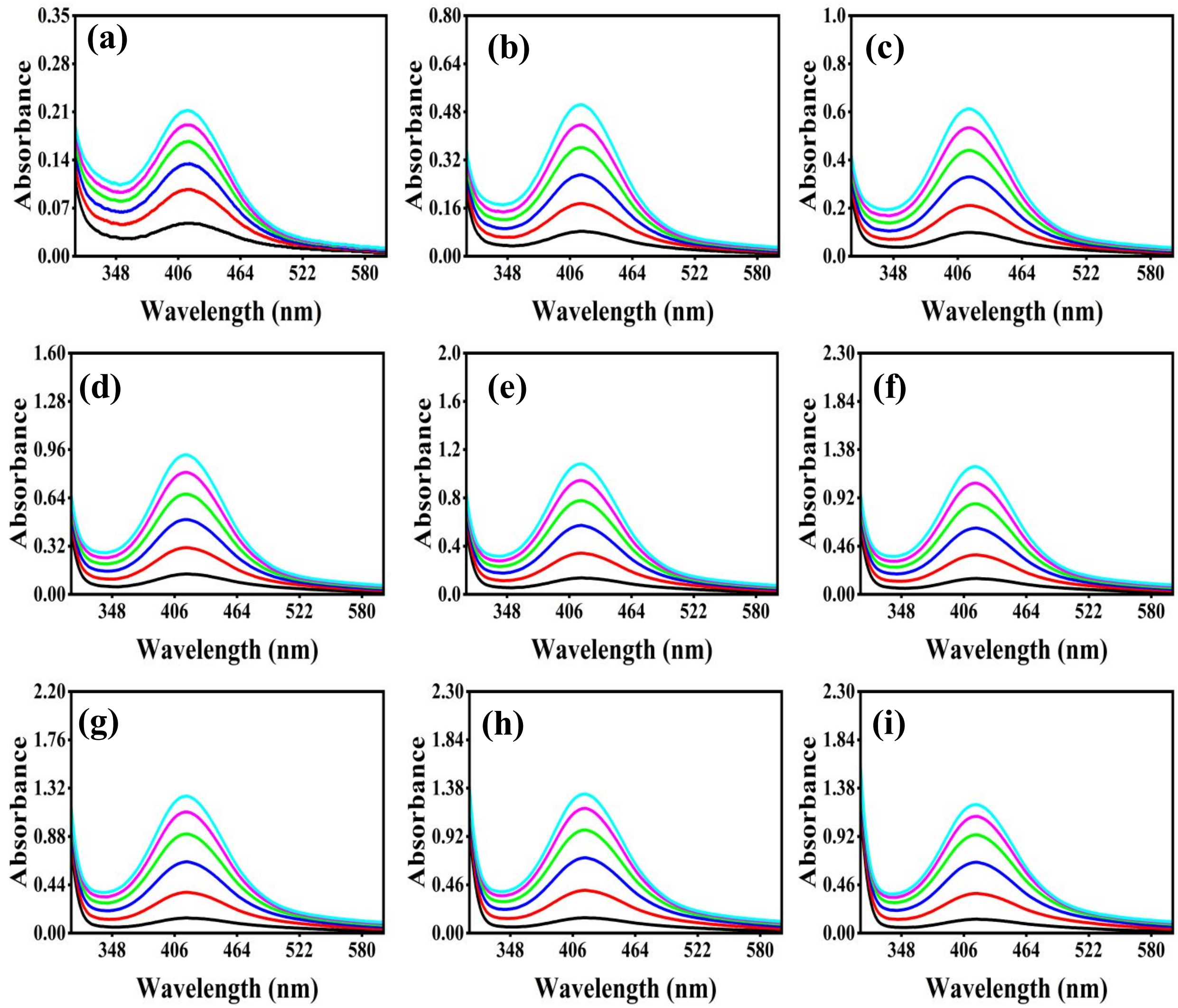

Figure S06. Time-dependent UV–visible absorption spectra recorded for 12 min. at an interval of 02 minutes at varying conc. of OPD (a) 0.1mM (b) 0.3 mM (c) 0.4 mM (d) 0.8 mM (e) 1.2 mM (f) 1.5 mM (g) 2 mM (h) 3 mM (i) 4 mM by keeping constant conc. of H_2O_2- 15 µL of 30% w/w and Cu@MCMCal- 50 µg/mL .

SI07. Time dependent UV–visible absorption spectra for varying conc. of H_2O_2 keeping OPD constant.

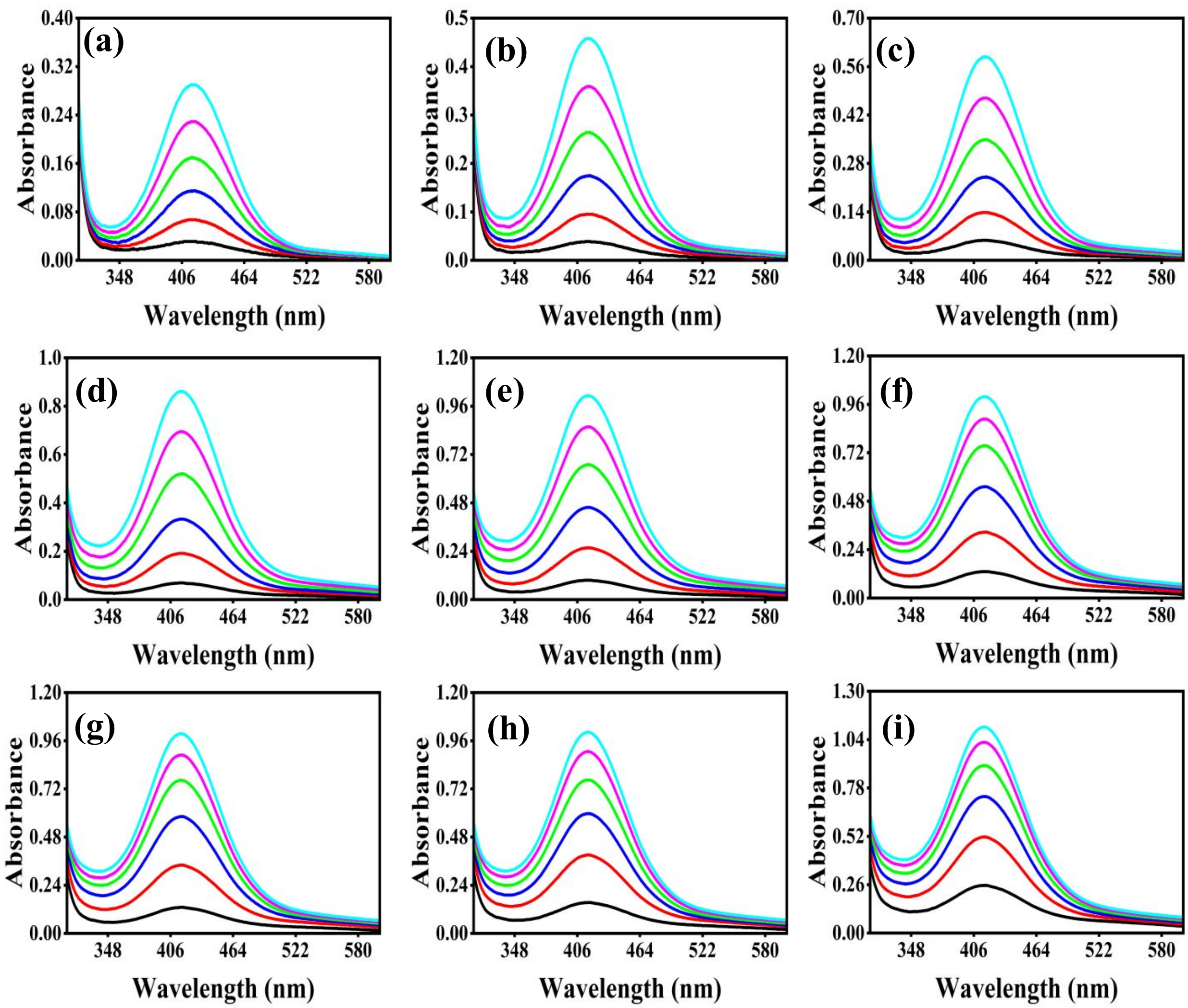

Figure S07. Time-dependent UV–visible absorption spectra recorded for 12 min. at an interval of 02 minutes at varying conc. of H_2O_2 (a) 0.33 mM (b) 0.67 mM (c) 1 mM (d) 2 mM (e) 3 mM (f) 4 mM (g) 5 mM (h) 6 mM (i) 10 mM by keeping constant conc. of OPD- 0.5 mM and Cu@MCMCal- 50 μg/mL .

SI08. PXRD and XPS of recycled Cu@MCMCal and UV-Vis absorption spectra for compare the peroxidase activity of different nanohybrid formed and scavenging experiment.

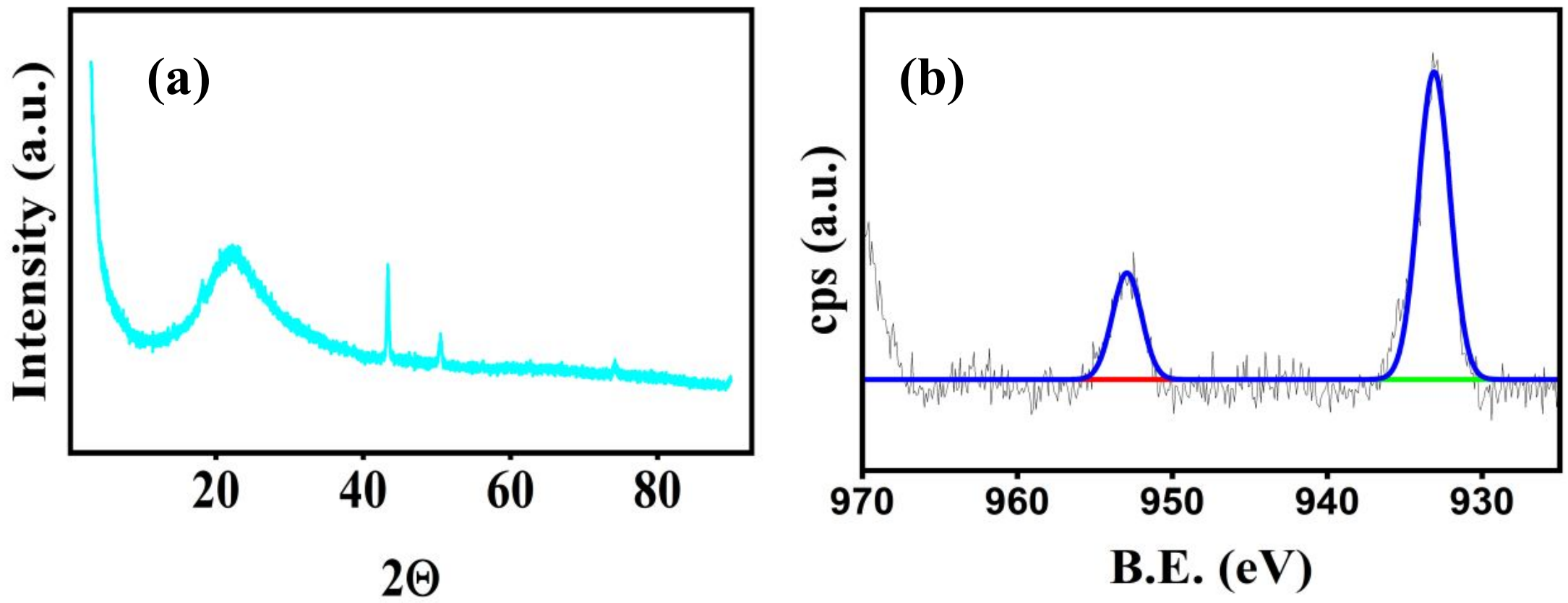

Figure S08. (a) PXRD of recycled Cu@MCMCal nanoenzyme and (b) XPS of recycled Cu@MCMCal nanoenzyme.

SI09. UV-Vis absorption spectra for compare the peroxidase activity of different nanohybrid formed and scavenging experiment.

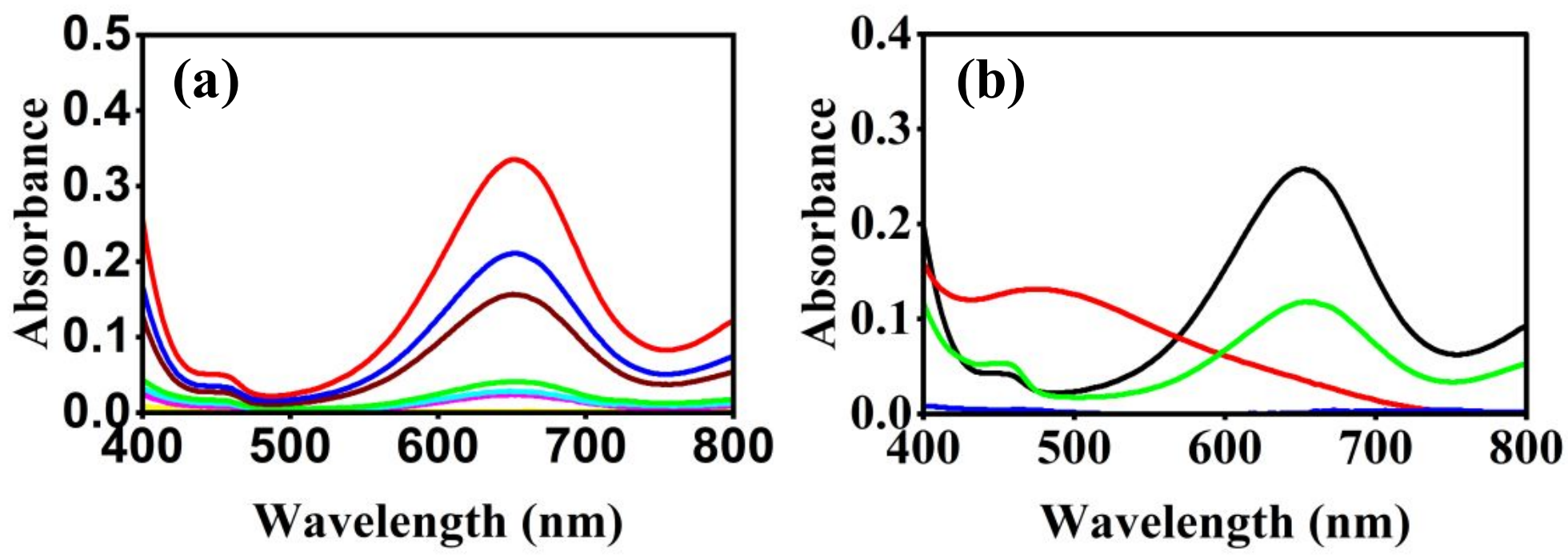

Figure S09. (a) Comparison of peroxidase activity: Relative absorbance at λ_{max}=652 nm for TMB oxidation in the presence of only catalyst (yellow, no oxidant); only H_2O_2 (magenta, no catalyst); MCM+H_2O_2 (cyan); MCMCal+H_2O_2 (green); CuNPs+H_2O_2 (wine); Cu@MCM+H_2O_2 (blue) and Cu@MCMCal+H_2O_2 (red) using TMB (0.5 mM), H_2O_2 (30 mM) and Cu@MCMCal-50 μg/mL and (b) UV–Vis spectra obtained from reactive oxygen species (ROS) scavenging experiments during the oxidation of TMB (0.2 mM) by H_2O_2 (25 μL, 30% w/w) catalyzed by Cu@MCMCal (50 μg/mL) nanomaterial after 12 min of reaction. The black curve represents the

complete reaction system without any scavenger. The green, red, and blue curves correspond to the reaction carried out in the presence of 10 μL of 99.5% IPA, PBQ (10 mM) and EDTA (10 mM), respectively.

SI10. Low-angle PXRD of MCM-41, MCMCal and Cu@MCMCal.

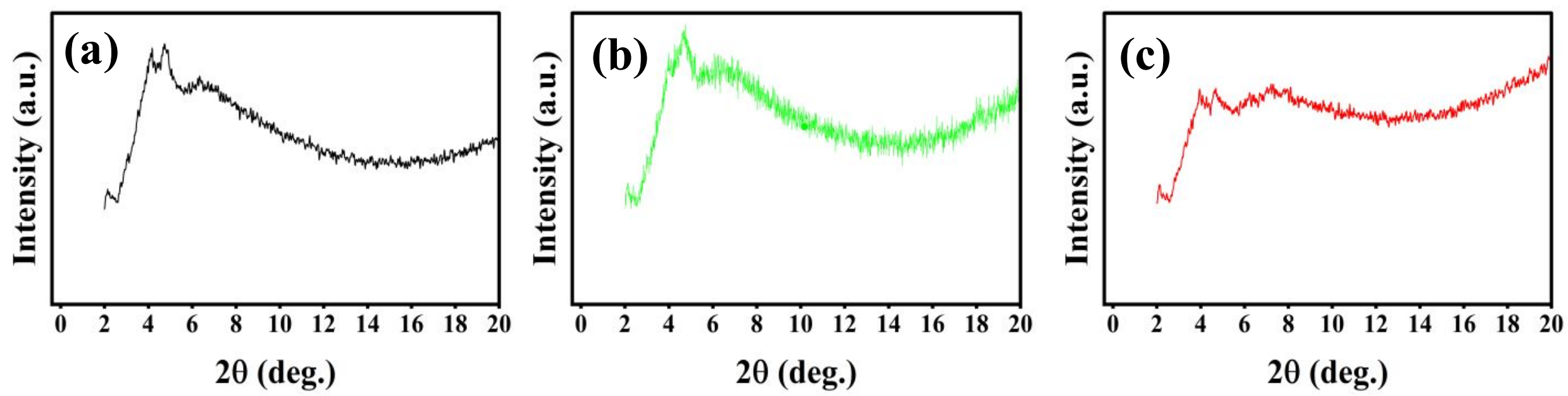

Figure S10. Low-angle PXRD: (a) MCM-41; (b) MCMCal; (c) Cu@MCMCal
